# Meta-analysis of metabolites involved in bioenergetic pathways reveals a pseudohypoxic state in Down syndrome

**DOI:** 10.1186/s10020-020-00225-8

**Published:** 2020-11-09

**Authors:** Laszlo Pecze, Elisa B. Randi, Csaba Szabo

**Affiliations:** Chair of Pharmacology, Section of Medicine, University of Fribourg, Fribourg, Switzerland

**Keywords:** Hypoxia, Glycolysis, Krebs cycle, Oxidative phosphorylation, Meta-analysis

## Abstract

Clinical observations and preclinical studies both suggest that Down syndrome (DS) may be associated with significant metabolic and bioenergetic alterations. However, the relevant scientific literature has not yet been systematically reviewed. The aim of the current study was to conduct a meta-analysis of metabolites involved in bioenergetics pathways in DS to conclusively determine the difference between DS and control subjects. We discuss these findings and their potential relevance in the context of pathogenesis and experimental therapy of DS. Articles published before July 1, 2020, were identified by using the search terms “Down syndrome” and “metabolite name” or “trisomy 21” and “metabolite name”. Moreover, DS-related metabolomics studies and bioenergetics literature were also reviewed. 41 published reports and associated databases were identified, from which the descriptive information and the relevant metabolomic parameters were extracted and analyzed. Mixed effect model revealed the following changes in DS: significantly decreased ATP, CoQ10, homocysteine, serine, arginine and tyrosine; slightly decreased ADP; significantly increased uric acid, succinate, lactate and cysteine; slightly increased phosphate, pyruvate and citrate. However, the concentrations of AMP, 2,3-diphosphoglycerate, glucose, and glutamine were comparable in the DS vs. control populations. We conclude that cells of subjects with DS are in a pseudo-hypoxic state: the cellular metabolic and bio-energetic mechanisms exhibit pathophysiological alterations that resemble the cellular responses associated with hypoxia, even though the supply of the cells with oxygen is not disrupted. This fundamental alteration may be, at least in part, responsible for a variety of functional deficits associated with DS, including reduced exercise difference, impaired neurocognitive status and neurodegeneration.

## Down syndrome as a metabolic disease: an introduction

Down syndrome (DS; also known as Down's syndrome or trisomy 21), is the most common genetic disorder known to date. It is caused by the presence of all or part of an additional (third) copy of chromosome 21. Its incidence is estimated to be approximately 1:700–1:1000 births worldwide. In the United States, approximately 6000 babies are born each year with DS (Bull 2020; Antonarakis et al. 2020).

Three different types of DS can be distinguished. The most common form of DS (approx. 95% of cases) features a whole and separate extra copy of chromosome 21. Translocation represents a rare form of DS (approx. 3% of cases), where the extra chromosome 21 (or part of it) is attached to another chromosome. Approximately 1% of DS cases are “mosaic DS” where the subject contains a mixture of two types of cells, some containing 46 chromosomes and some containing 47 (which contain the extra chromosome 21) (Bull 2020; Antonarakis et al. 2020).

The cause of the extra full or partial chromosome remains unknown, but increased maternal age is known to markedly increase the chance of DS. For instance, DS incidence increases from 1:1300 at age 25 years to 1:25 at age 49 years. Nevertheless, approximately 80% of children with DS are born to women who are younger than 35 years of age (Bull 2020; Antonarakis et al. 2020).

Subjects with DS can exhibit diverse pathophysiological alterations and deficits, ranging from mild to severe. Developmental alterations are common (phenotypically manifesting in physical growth delays and characteristic facial features, but also congenital heart disease and neurodevelopmental problems) and a progressive intellectual disability (Bull 2020; Antonarakis et al. 2020; Lagan et al. 2020). At later stage of life, DS produces an Alzheimer-syndrome-like state; this is so common that recent classifications consider DS as a congenital form of Alzheimer’s disease. A variety of additional conditions are also more common in DS, including airway, pulmonary and hearing disorders and hematological, oncological and autoimmune diseases (Bull 2020; Antonarakis et al. 2020; Lagan et al. 2020).

The scientific literature related to DS pathogenesis is massive, with significant bodies of literature focusing on the molecular genetics of the condition as well as on the pathogenesis and the potential experimental therapy of the neurocognitive issues, which are generally considered the most significant problems limiting the life of individuals with DS (Capone et al. 2006; Rachidi and Lopes 2010; Grieco et al. 2015; Malegiannaki et al. 2019; Lukowski et al. 2019; Martínez-Cué and Rueda 2020; Urbanus et al. 2020). However, there is also a significant body of clinical observations indicating that DS is associated with fundamental metabolic alterations, which, in turn, produce phenotypical changes, as well as increased propensities to various metabolic diseases.

For instance, DS individuals have less-than-normal muscle strength (muscle hypotonia) in their hands and lower extremities (Cioni et al. 1994; Croce et al. 1996; Carmeli et al. 2002; Hawli et al. 2009; Bala et al. 2018; Dupre and Weidman-Evans 2017; Coelho-Junior et al. 2019). They also exhibit significantly reduced aerobic exercise tolerance (Aguiar et al. 2008; Pastore et al. 2000). This has been commonly attributed to potential musculoskeletal developmental issues, neuromuscular junctional problems, as well as to the fact that DS individuals have decreased manual control, perform less physical activity and may have sarcopenia (Bala et al. 2018; Dupre and Weidman-Evans 2017; Coelho-Junior et al. 2019). While the above factors can certainly play a role, skeletal muscle functional analysis conducted in DS individuals (Phillips et al. 2013; Cowley et al. 2012) and data obtained from murine DS models (Cowley et al. 2012, 2017; Cisterna et al. 2014; Brault et al. 2015; Glass and Connor 2016; Bronks and Parker 1985) point to *an intrinsic bioenergetic and metabolic dysfunction* in the DS skeletal muscle. Importantly, a phosphorus magnetic resonance spectroscopy study in the quadriceps muscle of DS individuals indicates that the post-exercise resynthesis kinetics of phosphocreatine is suppressed (Phillips et al. 2013), which (a) indicates that intrinsic biochemical/bioenergetic mechanisms are impaired in the skeletal muscle of DS individuals and (b) suggests that the skeletal muscle’s mitochondrial respiratory function is suppressed in DS. Moreover, comparison of the contractile properties of soleus muscle of wild-type control mice and DS mice (Ts65Dn model) indicate that (despite the fact that soleus mass, skeletal mass:body weight ratio and muscle fiber type distribution are not different between control and DS mice), skeletal muscle contractions are impaired in DS (Cowley et al. 2012). While twitch and tetanus contractile responses were not significantly impaired in DS, the skeletal muscle from DS mice developed a progressive fatigue in repeated activation/recovery protocols (Cowley et al. 2012). The contractile weakness in DS skeletal muscle is associated with the dysregulation of multiple genes and gene products regulating various metabolic processes (including glucose uptake, lipid metabolism, oxidant/antioxidant balance), and has also been linked to mitochondrial dysfunction associated with DS (Hawli et al. 2009; Cowley et al. 2012, 2017; Cisterna et al. 2014; Brault et al. 2015; Glass and Connor 2016).

It is also well documented that many DS individuals present with an increased adiposity (Cowley et al. 2017; Bronks and Parker 1985; Rubin et al. 1998; Melville et al. 2005; Fonseca et al. 2005; González-Agüero et al. 2011; Soler Marín and Xandri Graupera 2011; Adelekan et al. 2012; Hill et al. 2013). This has been commonly attributed to the fact that DS individuals perform less physical activity and perhaps to different dietary habits (Hawli et al. 2009; Bala et al. 2018; Dupre and Weidman-Evans 2017; González-Agüero et al. 2011). Moreover, various lines of biochemical and endocrinology investigations, demonstrating increased adipokine production, altered lipid handling, subchronic inflammation, subclinical or manifest insulin resistance and/or metabolic syndrome (Magni et al. 2004; Corsi et al. 2009; de Asua et al. 2014a, b) and the development of fatty liver (Seeff et al. 1967; De Matteo and Vajro 2017) point to *intrinsic biochemical differences* in the development of obesity in DS.

If a generalized metabolic and bioenergetic dysfunction is present in DS, this would not only have the potential to underlie some of the above-mentioned more ‘classical’ metabolic alterations, but may also contribute to organ developmental problems, or even to neurological dysfunction (the neurons being highly ATP-dependent cells), and potentially the process of neurodegeneration as well (since cellular bioenergetic deficit can cause problems with protein processing) (see in more detail in “Potential mechanisms responsible for the development of pseudohypoxia in DS. Potential therapeutic interventions.” section). The fundamental role of metabolic and bioenergetic alterations in DS have been suspected for over 70 years and has periodically re-emerged as a working hypothesis (Simon et al. 1954; Anon 1954; Breg 1977; Shapiro 1983; Blass et al. 1988; Lejeune 1990; Chango et al. 2002; Coppedè 2009; Izzo et al. 2018; Vacca et al. 2019; Antonaros et al. 2020). Jerome LeJeune, the French physician-scientist, who discovered the chromosome abnormality in humans that causes DS, constructed a “Lejeune Machine” (Lejeune 1990), to illustrate the biochemical and metabolic alterations associated with this condition. However, the relevant scientific literature has not yet been systematically reviewed and organized to delineate the various metabolic alterations associated with DS. Therefore, in the current article, we present the results of our meta-analysis of metabolites involved in bioenergetics pathways in DS. First, we overview the basic cellular bioenergetic metabolic pathways, focusing on the primary as well as alternative sources that can yield ATP, the “universal energetic currency” in mammalian cells. Next, we present the results of our meta-analysis. Finally, we discuss potential underlying pathomechanisms and implications and review possible experimental therapeutic approaches.

## The principal bioenergetic pathways in mammalian cells: a brief overview

There are several metabolic pathways for the biotransformation of the fuel molecules to high energy compounds such as adenosine-5′-triphosphate (ATP), guanosine-5′-triphosphate (GTP), reduced nicotinamide adenine dinucleotide (NADH), reduced flavin adenine dinucleotide (FADH_2_) and reduced nicotinamide adenine dinucleotide phosphate (NADPH) (Dashty 2013). Glucose is the most important source of energy, but other monosaccharides, fatty acids, ketone bodies, amino acids, and nucleosides can also be used as an energetic source in a cell-specific manner (Berg et al. 2008).

### Glycolysis

Glycolysis is an evolutionarily ancient process of releasing energy from sugar which is already present in prokaryotes. In eukaryotic cells, glycolysis takes place in the cytosol. The essence of the process is that glucose is split into two molecules of pyruvate. Besides glucose, fructose and galactose can also enter the glycolytic pathway. This multistep process yields two ATP molecules and two molecules of NADH per molecule of glucose. In aerobic glycolysis, pyruvate is converted to acetyl-CoA so it can enter the mitochondria to be involved in the Krebs cycle (also known as “citric acid cycle” or “Szent-Györgyi-Krebs cycle”). However, in an anaerobic state, pyruvate remains within the cytoplasm and converts to lactate. The two molecules of NADH that are produced in glycolysis are oxidized to NAD^+^ by the reduction of pyruvate to lactate. Hence, anaerobic glycolysis produces only two molecules of ATP. Unused glucose molecules are packed and stored in the form of glycogen serving as a fast energy resource in the liver and in the muscle (Melkonian and Schury 2019).

### The pentose phosphate pathway

The pentose phosphate pathway is parallel to glycolysis. It occurs exclusively in the cytoplasm. The primary purpose of this alternative pathway is the generation of NADPH and the production of ribose-5-phosphate, to be used in the synthesis of nucleotides. However, this pathway is also involved in the catabolism of nucleosides, releasing energy from ribose molecules as pentose phosphate pathway is interconnected to the glycolysis through the shared used of glyceraldehyde-3-phosphate and fructose-6-phosphate (Aziz and Mohiuddin 2020).

### The Krebs cycle

The acetyl-CoA molecule inside the mitochondria is fully oxidized to carbon-dioxide. The total yield of one cycle is one molecule of GTP, three molecules of NADH, and one molecule of FADH_2_. NADH and FADH_2_ are reduced electron carriers, which donate electrons to the mitochondrial electron transport chain, while GTP is readily converted to ATP by a nucleoside-diphosphate kinase enzyme (Haddad and Mohiuddin 2019).

### Oxidative phosphorylation

The NADH and FADH_2_ donated-electrons reach the mitochondrial electron transport chain in the inner mitochondrial membrane and generate an electron flow through the mitochondrial Complexes (I, II, III and IV) thus pumping protons into the inner membrane space from the mitochondrial matrix. The resulting proton gradient is then used by the ATP synthase (also known as Complex V) to generate ATP molecules from ADP and inorganic phosphate. Oxidative phosphorylation produces 24–28 ATP molecules per glucose equivalent, more ATP than any other part of the cellular respiration (Haddad and Mohiuddin 2019).

### Beta oxidation of lipids

Fats provide an efficient means for storing energy for later use. Fats store more energy than glycogen and serve as a “slow energy resource”. Fatty acids are broken down into acetyl-CoA molecules through beta oxidation inside the mitochondria. Throughout each cycle of beta-oxidation, the fatty acid is reduced by two carbon lengths, producing one molecule of acetyl-CoA, and one molecule each of NADH and FADH_2_. The acetyl-CoA molecule can be oxidized in the Krebs cycle, while reduced electron carriers transfer their high energy electron to the mitochondrial electron transport chain (see above). Moreover, acetyl-CoA can be converted to ketone bodies. Ketone bodies including acetoacetate, beta-hydroxybutyrate, and acetone are produced by the liver during periods of glucose depletion. Ketone bodies are transported into organs requiring energy and converted back into acetyl-CoA, which then enters the Krebs cycle (Dunn and Grider 2020).

### Amino acid catabolism

Amino acids can also be used as a source of energy especially in times of starvation or high-protein content diet. The processing of amino acids results in the generation of intermediates of the citric acid cycle, including pyruvate, acetyl CoA, oxaloacetate, and α-ketoglutarate. In human blood, glutamine is the most abundant free amino acid. Glutamine catabolism, as an energy source, plays an important role in cancer cell survival (Jiang et al. 2019) and in the survival of mutant cells that harbor oxidative phosphorylation defects (Chen et al. 2018). Amino acid catabolism results in ammonia, a toxic byproduct. Ammonia can be detoxified by conversion to urea via the urea cycle, which occurs mainly in the liver. Urea produced by the liver is ultimately excreted by the kidney (Wu 2009).

### Pseudohypoxia

This term “pseudohypoxia” was originally coined by Ron Tilton’s group (Williamson et al 1993), referring to hypoxia-like change in the metabolic phenotype of cells in diabetes. In general, pseudohypoxia refers to a state in which cells or tissues express hypoxia-related genes and proteins and use re-wired metabolic pathways even when there is enough oxygen present. Pseudohypoxic phenotypes have been observed in a number of pathophysiological conditions, including sepsis (Tannahill et al. 2013) and Alzheimer’s disease (Salminen et al. 2017); it is also considered a component of physiological aging (Verdin 2015). More than a century ago Warburg (Warburg et al. 1927) observed similar phenomenon in various tumor cells; even when the cells are aerobic, they tend to mobilize glycolysis (often not as an alternative, but as a supplementary process to the normal oxidative phosphorylation) to maximize ATP generation that the cancer cells require for their fast division and multiplication.

## Gene expression, proteomic and metabolomic profiling of DS as it relates to cellular bioenergetics

Understanding the alterations in molecules provides important information for determining the molecular mechanisms of diseases. “Omics” technologies permit the universal detection of genes (genomics), mRNA (transcriptomics), proteins (proteomics) and metabolites (metabolomics) in biological samples (Ishihara and Akiba 2017). In DS, the genes encoded on chromosome 21 are exclusively up-regulated (and none of them are downregulated), supporting the “gene dosage theory” of DS i.e. that the extra chromosome 21 results in the upregulation of its gene products, which, in turn, directly or indirectly changes development and affects various cellular functions. However, approximately 90% of the differentially expressed genes in DS (which include both upregulated and downregulated genes) are encoded on chromosomes other than chromosome 21, indicating dysregulated signaling cascades and/or compensatory gene regulation mechanisms (Letourneau et al. 2014; Araya et al. 2019; Bally et al. 2020). Integration of different “omics” datasets (for example transcriptomics and proteomics) may provide a tool to link different networks, highlighting the cellular response to a perturbation (although, to our knowledge, in DS, several different types of omics approaches have not yet been systematically integrated). Nevertheless, “omics” approaches have many limitations. For instance, mRNA expression level is not always a good indicator for protein expression level (e.g. due to post-transcriptional regulation of protein stability)*,* and higher protein expression level does not necessarily mean a higher rate of an enzymatic reaction (for instance, because protein function is often regulated by post-transcriptional modifications, as well as by substrate and co-factor availability). In the following section, we will focus on the meta-analysis of metabolomic alterations, which represent the various products and substrates of enzymatic biochemical reactions; analysis of these metabolites may be a more direct indication of a metabolic pathway alteration than changes in mRNA or protein expression.

### Analysis methods used

#### Data collection and identification of studies

Journal articles published before July 1, 2020, were identified by using the search terms “Down syndrome” and “metabolite name” or “trisomy 21” and “metabolite name”. We checked reference lists and published review articles for additional potentially relevant studies. Moreover, metabolomics studies on human DS cells or individuals with DS were also collected.

#### Inclusion and exclusion criteria

The inclusion criteria for the present study were as follows: (1) We only included studies that measured the amount of the selected metabolites. (2) Studies had to include both a sample from DS individuals and a control comparison group (3) Studies had to provide means and standard deviations and information about sample sizes for both groups. Studies were also included, if they do not provide directly the means and standard deviations, but they can be estimated from the published data. (4) Studies providing data for the selected metabolites in different tissues or using different methods were all included. However, for measurements involving ATP or other high-energy phosphates, only *direct* measurements of total cellular high-energy phosphates were considered. (5) Only human samples were considered (data derived from animal models of DS were excluded). (6) Studies or experiments measuring metabolite levels after a treatment were excluded, similarly if the measurement was performed on cell lines at high (> 7) passage number. (7) Only publicly available datasets were used. In total 41 published reports and associated databases (all of which used cross-sectional study design) were identified and subjected to analysis.

### Variables included in data analysis

The following variables were collected: (1) The first author. (2) Year of publication. (3) The chemical name of the selected metabolite. (4) Mean of metabolite content in DS group. (5) Standard deviation of metabolite content in DS group. (6) Sample size in DS group. (7) Mean of metabolite content in control group. (8) Standard deviation of metabolite content in control group. (9) Sample size in the control group.

### Meta-analytic procedures

A meta-analysis was conducted with the *meta* R package (Balduzzi et al. 2019). Raw data from metabolomics experiments often contain unwanted technical variations between samples and data are typically right-skewed within a sample. In order to meet normality assumptions of several parametric tests, data are usually log2 transformed. Technical variations such as signal drifts or batch effects are frequently encountered in metabolic profiling thus removing these effects by normalization is now widely considered as an integral part of data processing (Li et al. 2017). Several methods are available, but still, sample normalization is sometimes ignored in metabolomics studies. We used log2 transformation if authors reported statistics on skewed data and variance stabilizing normalization (Huber et al. 2002), if the authors did not normalize, but it would have been necessary. Further details can be found in the Supplementary Material. Adjustment for potential confounders, (e.g. age or sex) was not performed, and most of the cases these data were not available on individual levels.

The following studies give directly group means (M), standard deviations (SD) and sample sizes (N): Helguera et al. (2013), Bayer and McCoy (1974), Bartels (1971, Knull et al. (1978), Puukka et al. (1982), Kedziora et al. (1972), Nelson and Benson (1972), de Asua et al. (2014a,b), Lejeune et al. (1992), Nura et al. (2008), Infantino et al. (2011), Zitnanova et al. (2004), de Sousa et al. (2015); Howell et al. (1973); Kaufman and O’Brien (1967), Campos et al. (2010), Yates et al. (1990), Zaki et al. (2017), Coppus et al. (2007), Pogribna et al. (2001) and Miles et al. (2007).

If the standard error of the mean (SEM) is reported, standard deviations can be derived by using the formula: SD = SEM*sqrt(N). This conversion was performed on the following studies: Stocchi et al. 1985, Valenti et al. 2010, Rodríguez-Sureda et al. 2015, Izzo et al. 2017, Heggarty et al. 1996, Watkins et al. 1989, Tiano et al. 2008, and Convertini et al. 2016.

In some studies, the values of data were plotted in graphs. In this situation, values were extracted using *digitize* R package (Poisot 2011) or Adobe Acrobat Reader measurement tools.

Mircher reported age-segmented data (Mircher et al. 1997). In this case, age-segmented data were sequentially combined according to the Cochrane textbook (Higgins and Green 2008). Being N the number of individuals in a given segment, M the mean and SD the standard deviation of segments 1 and 2, mean is calculated as (N1*M1 + N2*M2)/(N1 + N2). For SD, it is *the square root of*: [(N1 − 1) SD1^2^ + (N2 − 1) SD2^2^ + ((N1*N2/(N1 + N2)) (M1^2^ + M2^2^ − 2*M1*M2))]/(N1 + N2 − 1). Similarly, age and gender groups were combined for the study of Pant and colleagues (Pant et al. 1968).

Chapman and Stern (1964) and Rosner et al. (1965) reported mean values and ranges. SD from ranges was calculated using the conversion factor provided by the study of Walter and Yao (2007).

Caracausi et al. (2018) and Antonaros et al. (2020) reported raw data values. M, SD, and N were calculated for each compound both in DS and in control groups, as well as in plasma and in urine samples while the fasting condition was not considered. More details can be found in Additional file 1.

Culp-Hill et al. (2017) reported untargeted and targeted metabolomics datasets. Data were filtered for the age: 12 ≤ Age ≤ 54, in line with the original study. Normalization and log2 transformation were performed on the raw data. If the compound is present both in the targeted and the untargeted compound list, only the targeted values were considered. More details can be found in Additional file 1.

In the study of Powers et al. (2019) we used the adjusted_met_data dataset, to calculate M, SD and N for a given metabolite in DS and control plasma.

Orozco et al. (2019) reported regression coefficients from multiple linear regression performed for each metabolite to assess the association between neurodevelopmental diagnosis (independent variables) and plasma metabolites (dependent variable). Typical development (control) children were used as a reference group. Adjusting for sex, race/ethnicity, age, year of blood collection, and parental homeownership was performed. From the published parameters i.e. betas, confidence intervals (95%CI_max_ and 95%CI_min_), and sample sizes (NDS for DS, and NC for control), we calculated the M and SD for meta-analysis as M (for DS) = beta, M (for control) = 0, SD (for both) = ((95%CI_max_ − 95%CI_min_)/(2*t))/sqrt(1/N_DS_ + 1/N_C_), when t is the t-value for the given probability (0.975) and degree of freedom. The degree of freedom for multiple regression is calculated as n − k − 1, where n = N_DS_ + N_C_, and k refers to the number of regressors not including the intercept.

Obeid et al. (2012) reported median, 10% quantile, and 90% quantile. In this case, the group mean was estimated as log2(median), while SD was estimated as [log2(90%quantile)-log2(10%quantile)]/(2*z0.9), where z0.9 = 1.28.

Fuller et al. (1962) and Mertz et al. (1963) reported individual data, from which M, SD and N were calculated.

A random-effects model (Borenstein et al. 2011) was used to determine the expected high degree of heterogeneity across studies using Hedges’ g as the standardized mean difference (Hedges 1981). Besides that, the 95% confidence intervals (CI) were estimated. Positive and negative SMD values indicated higher and lower levels of metabolites in DS group, relative to the control group. Statistical significance was set at p < 0.05.

Forty-one studies met the inclusion criteria, were critically reviewed, and are summarized in Table 1.

**Table 1:** Studies selected for meta-analysis

Study	# of DS	# of CTR	Analytes measured	Biological matrix
Antonaros et al. (2020)	129	46	Citrate, glucose, glutamine, lactate, pyruvate, succinate, tyrosine	Plasma
Bartels (1971)	15	15	ATP, ADP, AMP	RBC
Bayer and McCoy (1974)	8	19	ATP, ADP, AMP	Platelet
Campos et al. (2010)	38	28	Uric acid	Urine
Caracausi et al. (2018)	51	20	Citrate, tyrosine	Plasma, urine
Chapman and Stern (1964)	40	40	Uric acid	Serum
Convertini et al. (2016)	3–6	3–6	Citrate	Lymphoblast, PBMC
Coppus et al. (2007)	46	48	Tyrosine, serine	Plasma
Culp-Hill et al. (2017)	29	43	Citrate, glucose, lactate, pyruvate, serine, succinate, tyrosine, uric acid, 2,3-DPG, ADP, AMP, arginine, ATP, glutamine, homocysteine, Pi	RBC
de Asua et al. (2014a,b)	48	33	Glucose	Plasma
de Sousa et al. (2015)	30	30	Uric acid	Saliva
Fuller et al. (1962)	80	80	Uric acid	Serum
Heggarty et al. (1996)	21–22	18–20	Arginine, cysteine, glutamine, serine, tyrosine	Plasma, urine
Helguera et al. (2013)	10	10	ATP	Neuron
Howell et al. (1973)	113	106	Uric acid	Serum, urine
Infantino et al. (2011)	6	6	Cysteine, serine	WBC
Izzo et al. (2017)	5	4	ATP	Fibroblast
Kaufman and O’Brien (1967)	107	107	Uric acid	Serum
Kedziora et al. (1972)	6	7	ATP, ADP, AMP, Pi, 2,3-diphosphoglycerate	RBC
Knull et al. (1978)	19	22	ATP, 2,3-diphosphoglycerate	RBC
Lejeune et al. (1992)	79	206	Serine, cysteine, tyrosine, arginine	Plasma
Mertz et al. (1963)	25	25	Uric acid	Serum
Miles et al. (2007)	14	12	CoQ10	Plasma
Mircher et al. (1997)	107	216	Serine, tyrosine, arginine	Plasma, urine
Nelson and Benson (1972)	20	20	2,3-diphosphoglycerate	RBC
Nura et al. (2008)	48	37	Serine, glutamine, arginine	Plasma
Obeid et al. (2012)	35	47	Cysteine, homocysteine	Plasma, ferum
Orozco et al. (2019)	31	193	Serine, lactate, pyruvate, tyrosine, glutamine, succinate, arginine	Plasma
Pant et al. (1968)	356	360	Uric acid	Plasma
Pogribna et al. (2001)	42	36–38	Cysteine, homocysteine	Plasma
Powers et al. (2019)	72–75	79–90	Citrate, glucose, arginine, cysteine, glutamine, homocysteine, serine, tyrosine, lactate, Pi, pyruvate, succinate, uric acid	Plasma
Puukka et al. (1982)	10	10	ATP, ADP, AMP	RBC
Rodríguez-Sureda et al. (2015)	5	5	ATP	Fibroblast
Rosner et al. (1965)	12	12	Pi, uric acid	Serum
Stocchi et al. (1985)	20	20	ATP, ADP, AMP	RBC
Tiano et al. (2008)	30	30	CoQ10, uric acid	Lymph, plasma, platelets
Valenti et al. (2010)	5	5	Lactate, ATP, ADP, AMP	Fibroblast
Watkins et al. (1989)	15–18	19	Serine, tyrosine, cysteine, arginine	Plasma
Yates et al. (1990)	6	29–30	Lactate	Caudate nucleus, frontal cortex
Zaki et al. (2017)	43	43	CoQ10, glucose	Plasma
Zitnanova et al. (2004)	16	16	Uric acid	Plasma

### Alterations in high energy molecule pools in DS cells and tissues

**Fig. 1:**
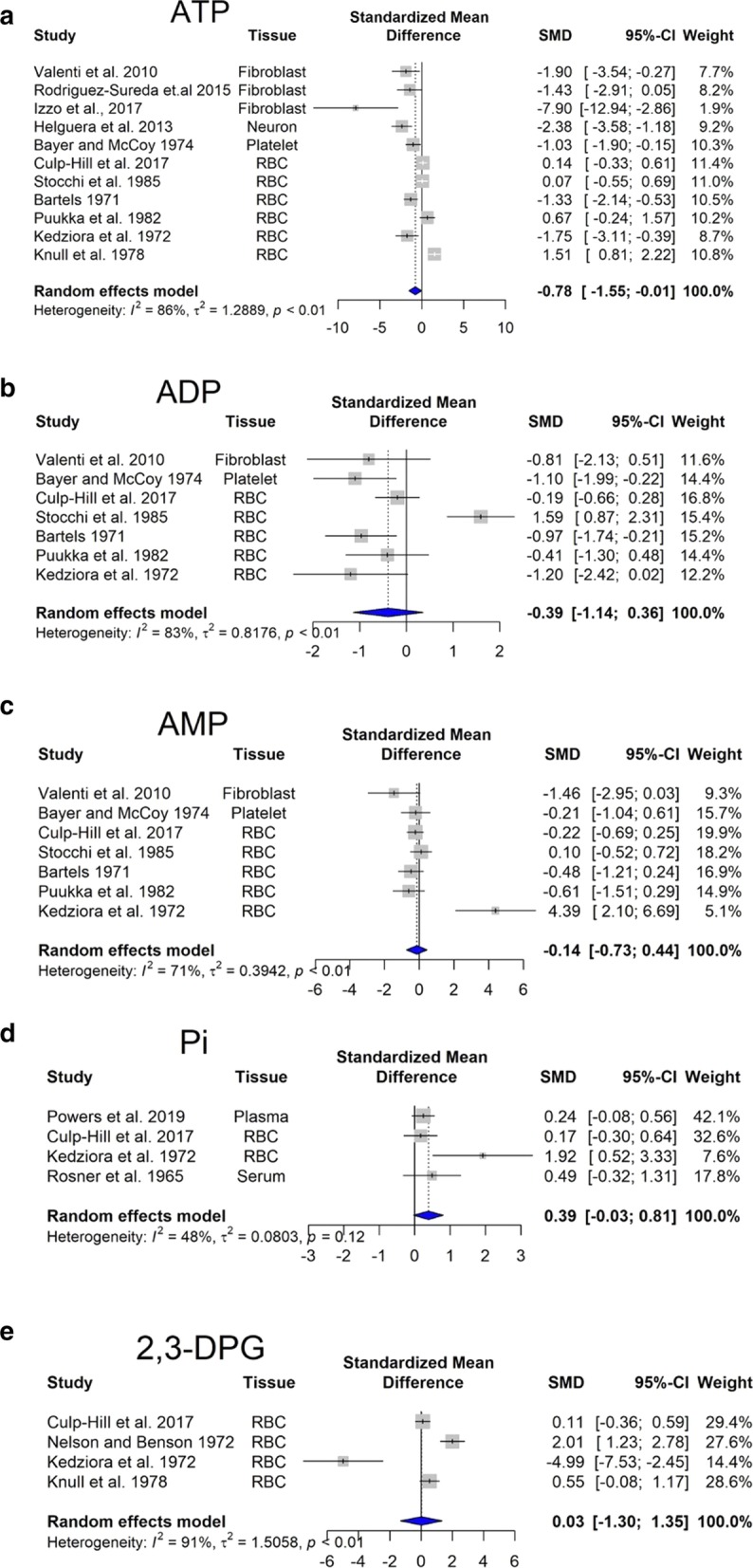
Changes in bioenergetics-related analytes in Down syndrome. Forest plot showing relative weights, standardized mean difference (Hedge’s g) with confidence intervals. Overall average effect size is displayed by filled diamond

Within cells, energy is provided by oxidation of fuel molecules such as carbohydrates, lipids, and proteins. The oxidation process results in free energy production that can be stored in high-energy bonds within molecules such as nucleoside triphosphate, phosphoenolpyruvate, carbamoyl phosphate, 2,3-bisphosphoglycerate, phosphoarginine, or phosphocreatine (Bonora et al. 2012). Kedziora and his coworkers have reported decreased levels of 2,3-diphosphoglyceric acid, and increased content of AMP, GTP, NAD^+^, and NADP^+^ (Kedziora et al. 1972). Our meta-analysis performed on ATP, ADP and AMP levels revealed statistically significant changes. Importantly, we observed that cellular ATP content is significantly lower in DS individuals (Fig. 1a), while ADP is slightly reduced (Fig. 1b) and AMP is unchanged (Fig. 1c). In addition, in DS individuals, there was a tendency for an increase in inorganic phosphate (Pi) levels (Fig. 1d). Similar trends in the composition of energy molecule pool have been observed in hypoxic state. In hypoxia, there is a fall in [ATP] and an increase in [ADP], along with an increase in Pi (Taggart and Wray 1998). These alterations resulted in a lower energy charge of DS cells compared to controls (see: Additional file 1).

The molecule 2,3-diphosphoglyceric acid (2,3-DPG) is very important in regulating the affinity of hemoglobin for oxygen. If the concentration of 2,3-DPG in red blood cell is low, hemoglobin affinity for oxygen is increased and tissue hypoxia may occur (MacDonald 1977). Our meta-analysis revealed no significant difference in the content of 2,3-DPG between DS and control red blood cells (Fig. 1e). This finding may indicate that oxygen delivery to DS tissues may not be impaired, but—based on the low ATP output, we hypothesize that—they may use less oxygen compared to healthy individuals (pseudo-hypoxia).

**Fig. 2:**
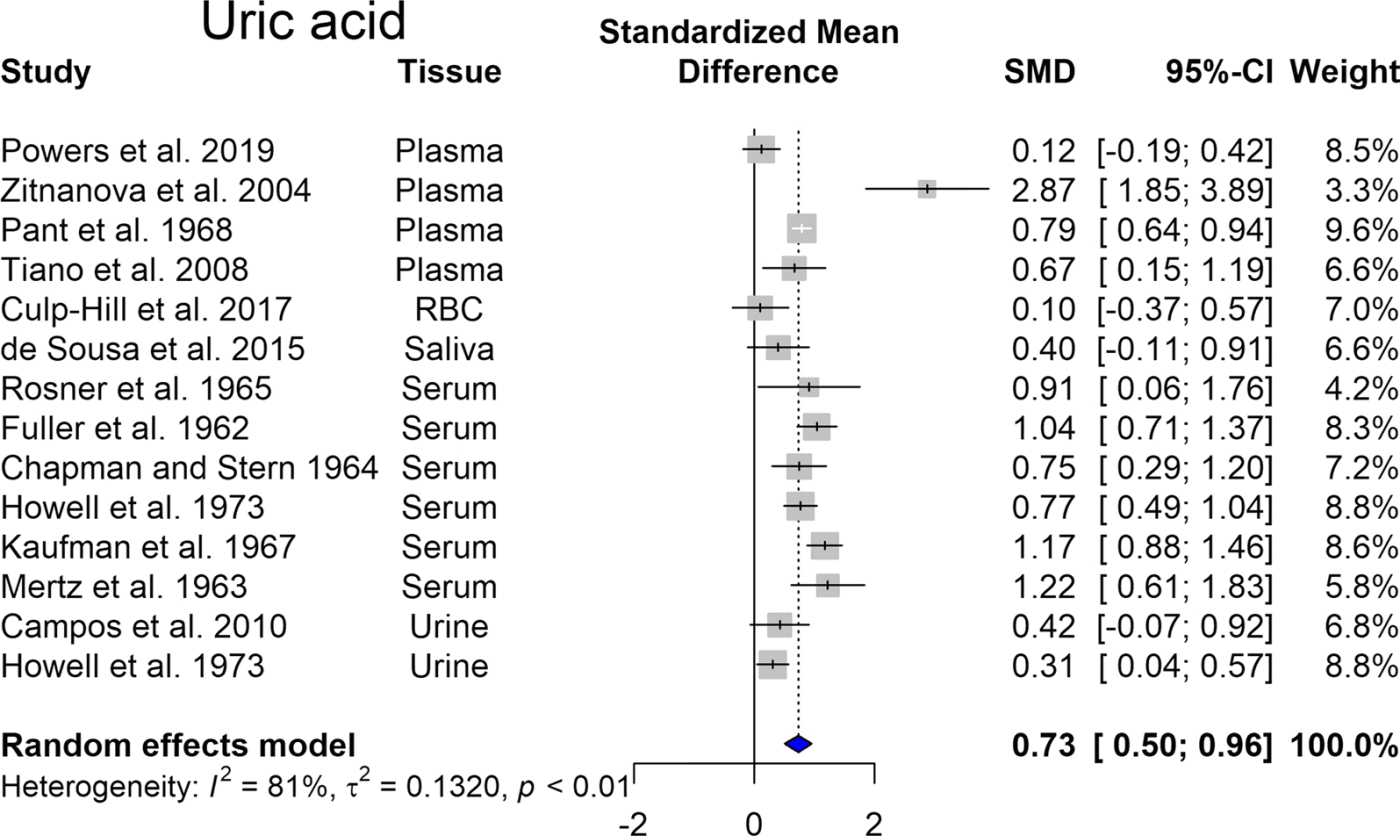
Changes in uric acid in Down syndrome. Forest plot showing relative weights, standardized mean difference (Hedges’ g) with confidence intervals. Overall average effect size is displayed by filled diamond

Uric acid (UA) is the end product of ATP and GTP catabolism and can reportedly act as an antioxidant. Several studies indicate elevated uric acid levels and increased prevalence of gout in DS (Igoe et al. 2017). In line with these findings, our meta-analysis showed that this elevation is statistically significant (p < 0.05) (Fig. 2). As higher urinary uric acid excretion is an indicator of severe hypoxia (Ozanturk et al. 2006), we can assume that a pseudo-hypoxic state develops in DS.

Further evidence for pseudohypoxia would be an increased NADH to NAD^+^ ratio (Gomes et al. 2013), but we have not found a study addressing this question in DS. Stocchi and colleagues reported that the total levels of nicotinamide adenine dinucleotide (NADH and NAD^+^) and nicotinamide adenine dinucleotide phosphate (NADPH and NADP^+^) were within normal ranges in DS individuals (Stocchi et al. 1985).

### Changes in mitochondrial electron transport/oxidative phosphorylation in DS

As already mentioned earlier, DS is characterized by an overexpression of genes on chromosome 21, in addition to an overall dysregulation of an even larger set of genes located on the other chromosomes (Letourneau et al. 2014; Araya et al. 2019; Culp-Hill et al. 2017; Mao et al. 2005). Among these, there are genes involved in oxidative phosphorylation (OXPHOS), including all five mitochondrial complex subunits, genes implicated in mitochondrial biogenesis, and more generally in the mitochondrial function. Microarray analyses of DS samples showed a marked downregulation of genes encoding mitochondrial proteins, called nuclear-encoded mitochondrial genes (Conti et al. 2007; Chen et al. 2012; Sullivan et al. 2016; Liu et al. 2017; Sobol et al. 2019; Lee et al. 2003) (Table 2).

**Table 2:**
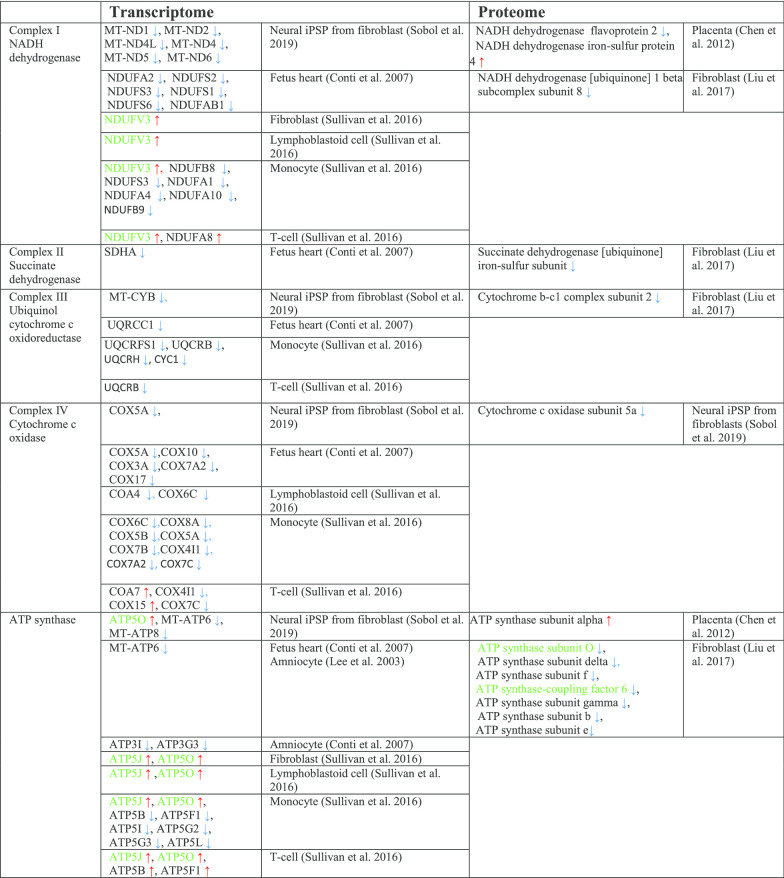
Changes in genes and proteins involved in oxidative phosphorylation in DS

Genes/proteins localized on chromosome 21 are shown in green color

The first study suggesting a functional association between impaired mitochondria and DS was conducted in 1994. Prince and colleagues reported a decreased activity of mitochondrial enzymes in platelets from DS individuals compared to normal controls (Prince et al. 1994). Subsequent studies demonstrate a reduction in mitochondria energy production efficiency in DS fibroblasts due to the impairment of complex I, V, ADP/ATP translocator and adenylate kinase activities (Schapira 1999; Piccoli et al. 2013). Additionally, the expression of several different mitochondrial electron transport enzyme subunits is decreased in the brain from DS individuals (Kim et al. 2001). DS fibroblasts are characterized by increased production of reactive oxygen species (ROS), higher intra-mitochondrial Ca^2+^ levels, and bioenergetic deficit, with decreased basal ATP content and lower mitochondrial membrane potential (Schapira 1999; Piccoli et al. 2013; Kim et al. 2001; Panagaki et al. 2019, 2020).

**Fig. 3:**
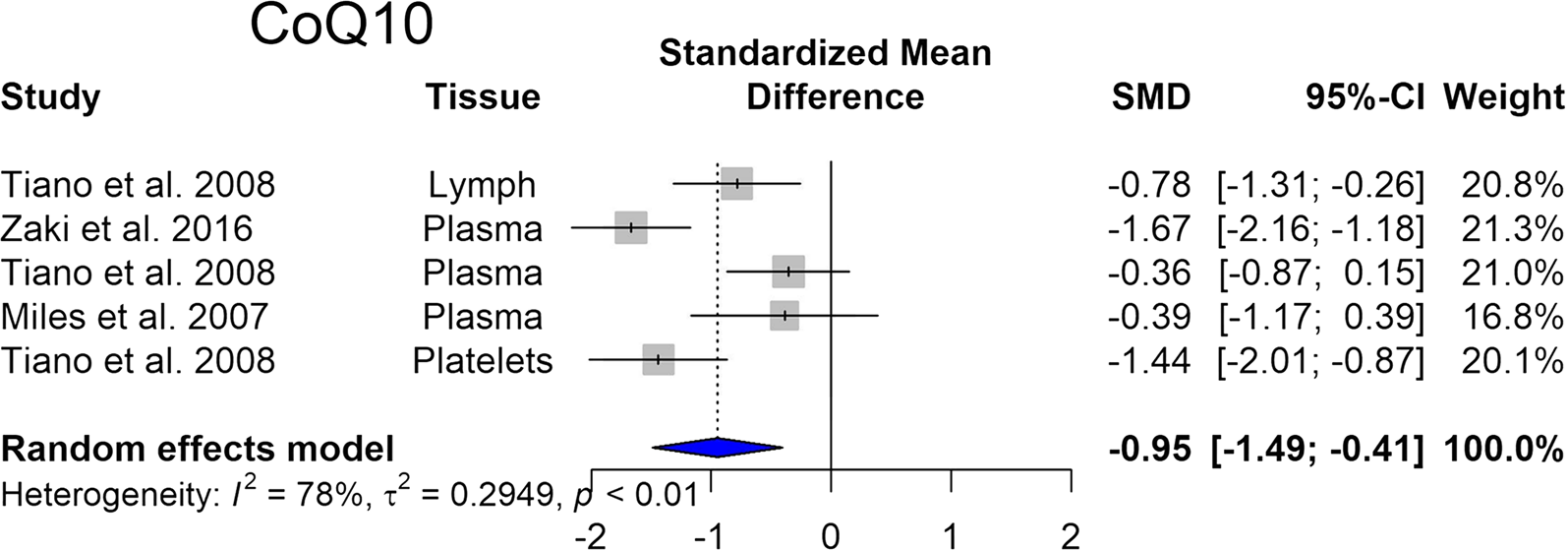
Changes in CoQ10 in Down syndrome. Forest plot showing relative weights, standardized mean difference (Hedges’ g) with confidence intervals. Overall average effect size is displayed by filled diamond

Coenzyme Q10 (CoQ10), a lipid molecule is an essential component of the electron transfer chain and it is an endogenous cellular antioxidant. CoQ10 takes part in aerobic cellular respiration and generation of energy in the form of ATP. Our meta-analysis reveals that the levels of CoQ10 are significantly reduced in DS (Fig. 3).

Electron microscopy analysis of fetal DS hearts provided a deeper understanding of mitochondrial abnormalities in DS. Most of the DS mitochondria were characterized by morphological alterations, such as increased fragmentation, bigger size, irregular shape, and cristae’s patterns (Schapira 1999; Piccoli et al. 2013; Kim et al. 2001; Panagaki et al. 2019, 2020). Similar changes have been reported in hypoxic conditions, namely increased ROS production, together with a decrease in the activity in mitochondrial complexes and mitochondrial fragmentation (Lee et al. 2020a, b).

### Glycolytic alterations in DS

If oxidative phosphorylation is impaired, NADH and FADH_2_ cannot be oxidized as rapidly in the electron transport chain and ATP cannot be generated. To maintain the energy supply of the cell*,* alternative pathways for producing energy are utilized.

Accordingly, there is an increased level of anaerobic glycolysis in DS. The phosphorylation of fructose 6-phosphate is irreversible, and phosphofructokinase, the enzyme that catalyzes it, is the rate-limiting enzyme in glycolysis (Mor et al. 2011). Several early reports show that there is an increase in the activity of phosphofructokinase enzyme in fibroblast and red blood cells derived from individuals with DS (Layzer and Epstein 1972; Baikie et al. 1965; Annerén et al. 1987; Conway and Layzer 1970). Nevertheless, the gene for the liver type subunit of phosphofructokinase is located on human chromosome 21 indicating a higher gene expression.

**Fig. 4:**
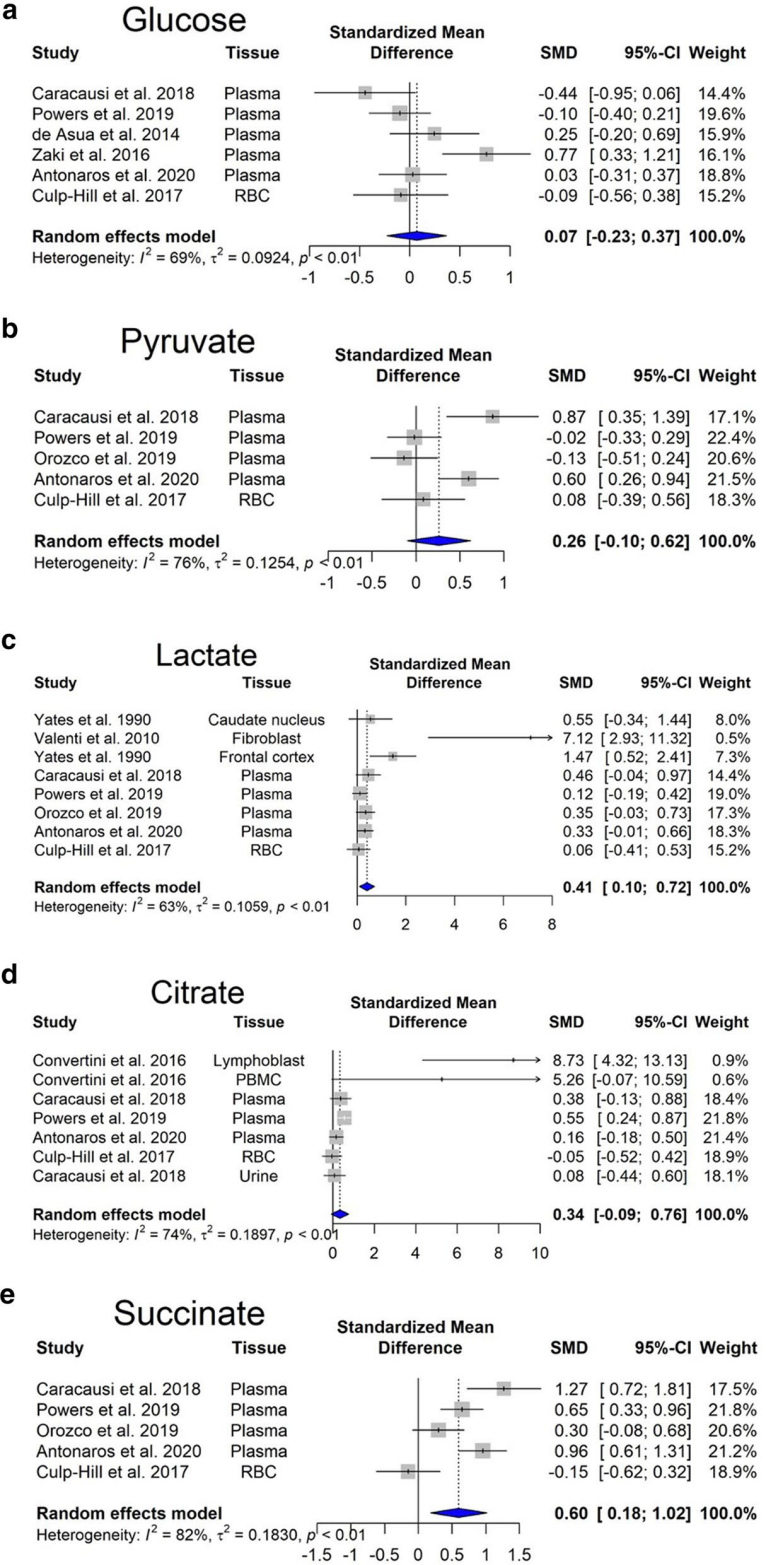
Changes in Krebs cycle-related analytes in Down syndrome. Forest plot showing relative weights, standardized mean difference (Hedges’ g) with confidence intervals. Overall average effect size is displayed by filled diamond

Overexpression of enzymes involved in glycolysis would predicts an increased glucose consumption of the DS cells. Our meta-analysis revealed no significant changes in glucose steady-state concentrations (Fig. 4a). However, there is an increasing trend in pyruvate concentration (Fig. 4b).

Glycolysis produces 2 ATP molecules and 2 NADH molecules. NADH generated during aerobic glycolysis cannot reach the electron transport chain directly. The malate-aspartate shuttle mechanism serves to move the reduced NADH across the membrane in the form of other reduced molecules (Nelson et al. 2008). However, in DS, the electron transport chain is directly inhibited {as discussed later, recent data suggest that one of the reasons is that Complex IV activity is suppressed by the increased hydrogen sulfide [H_2_S] production, resulting from increased cystathionine-beta-synthase [CBS] and 3-mercaptopyruvate sulfurtransferase [3-MST] expression in DS cells (Panagaki et al. 2019, 2020)}. Hence, NADH molecules remain in the cytosol and oxidized in the process of lactic fermentation to regenerate NADH molecules produced in glycolysis. Our meta-analysis revealed that lactate production, i.e. anaerobic glycolysis is significantly elevated (p < 0.05) (Fig. 4c) in Tamarkina et al. (1978) and Baikie et al. (1965) showed that lactate dehydrogenase activity in DS samples was not elevated, indicating an upstream regulation, most probably at phosphofructokinase level (Tamarkina et al. 1978). Increased blood lactate concentration is traditionally attributed to anaerobic glycolysis due to inadequate oxygen delivery (James et al. 1999).

### Changes in amino acid utilization in DS

Amino acids derived from the dietary proteins serve as an energy source as they can enter the citric acid cycle, while their ammonia moiety is eliminated through the urea cycle. Glutamine is the most abundant extracellular amino acid in the blood plasma, and it is quantitatively the most important fuel among the amino acids. Nevertheless, there are no changes in its concentrations between DS and control subjects (Fig. 4a). It is immediately metabolized to glutamate by glutaminase. Later, glutamate undergoes transamination with pyruvate generating l-alanine and 2-oxoglutarate (also known as alpha-ketoglutarate). The latter metabolite is then oxidized in the Krebs cycle generating succinate (Nelson et al. 2008). Energy molecules, NADH and GTP, are generated via this pathway. GTP can be easily converted to ATP in the cell (Conway and Layzer 1970). As in DS the mitochondrial electron transport chain is inhibited, the produced NADH may be oxidized in another manner.

One possible way is the reduction of alpha-ketoglutarate to 2-hydroxyglutarate by using NADH. L-2-hydroxyglutarate is found to be produced to high levels in low oxygen conditions, including cells of the immune system and cancer cells (Tyrakis et al. 2016). In DS plasma, Powers and colleagues demonstrated an increase in the levels of 2-hydroxyglutarate (Powers et al. 2019).

Second, mitochondrial isocitrate dehydrogenase can catalyze the reverse reaction converting alpha-ketoglutarate to isocitrate and then citrate (reductive carboxylation). During this step, NADH is oxidized and the resulting citrate is then transported out of the mitochondria, to the cytosol, where the enzyme citrate lyase converts citric acid to acetyl-CoA (Corbet and Feron 2015)*.* This is the initial step of de novo lipid biogenesis. Mitochondrial isocitrate dehydrogenase-mediated reductive carboxylation is observed when steady-state alpha-ketoglutarate levels are high, and that of citrate is low (Eales et al. 2016). In DS individuals, elevated levels of alpha-ketoglutarate have been found in plasma (Powers et al. 2019) and in red blood cells (Caracausi et al. 2018). Moreover, our meta-analysis showed a significant increase in citrate levels (Fig. 4d). In hypoxia, there is also a significant increase in the levels of molecules involved in the citric acid cycle, such as citrate, succinate, fumarate (Bénit et al. 2014; Peng et al. 2019).

Succinate produced during the citric acid cycle, is transported across the inner mitochondrial membrane by the mitochondrial dicarboxylate carrier and acts as a mediator of mitochondrial dysfunction (Tretter et al. 2016). Our meta-analysis revealed that succinate accumulates in DS (Fig. 4e). It has been shown that succinate stabilizes hypoxia-inducible factor 1-α, leading to hypoxia-like metabolic phenotypes in the absence of hypoxia, also known as pseudohypoxia. Succinate also inhibits histone and DNA demethylases changing the epigenetic profile (Tretter et al. 2016). Moreover, succinate might be involved in ROS generation, through Complex I (Chouchani et al. 2014). The presence of increased ROS levels and oxidative stress in DS individuals has been well established (Valenti et al. 2011).

**Fig. 5:**
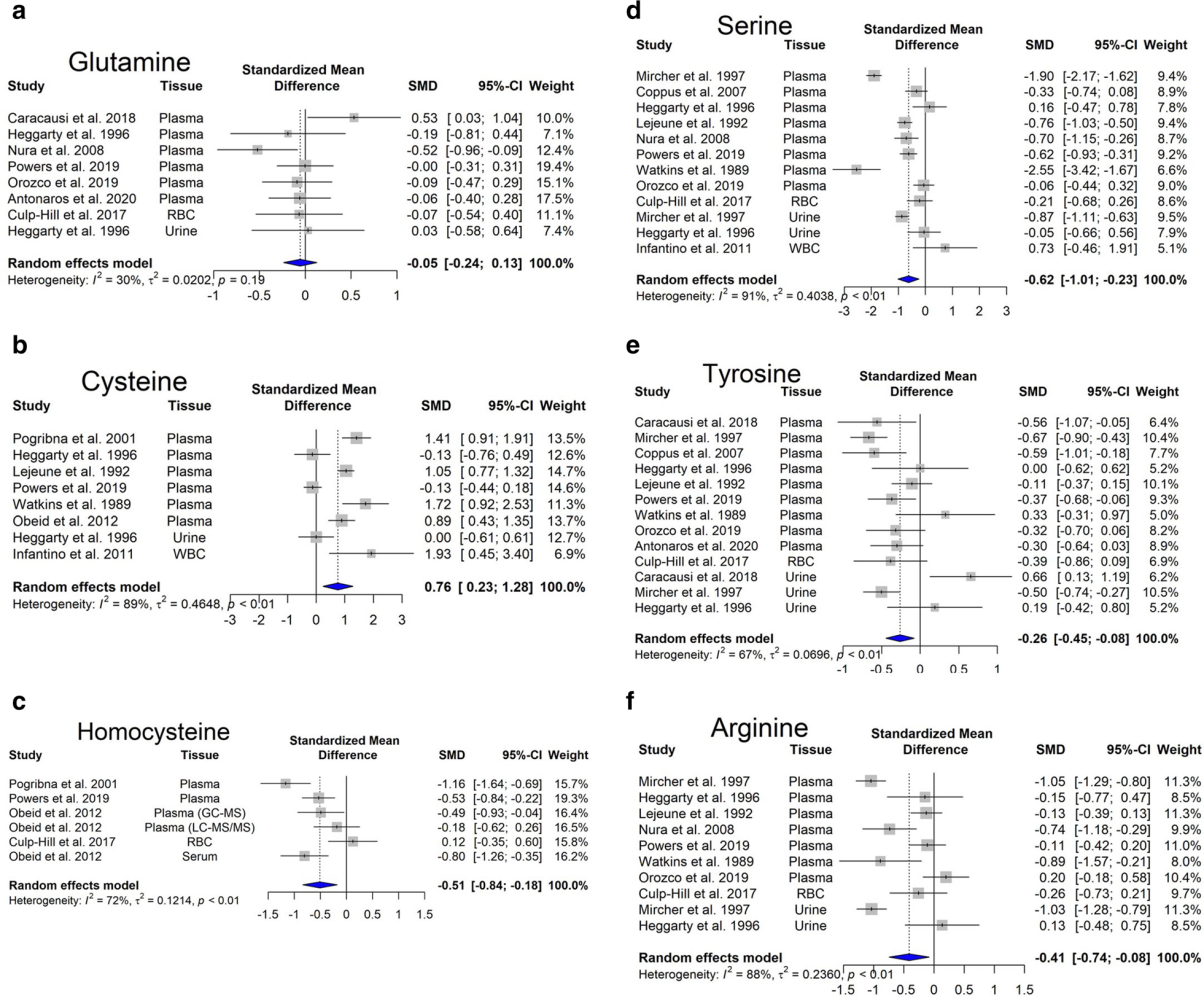
Changes in selected amino acids in Down syndrome. Forest plot showing relative weights, standardized mean difference (Hedges’ g) with confidence intervals. Overall average effect size is displayed by filled diamond

Besides that, several alterations on amino acid concentrations have been described in the blood of DS in comparison with control subjects (Fig. 5). The most consistent findings are the following: increased cysteine (Fig. 5b) and decreased serine (Fig. 5c) and homocysteine (Fig. 5d) levels (most probably due to a dosage effect of the gene for CBS), as well as decreased tyrosine and arginine levels, perhaps due to the pseudo-hypoxic state of DS cells. In fact, it is known that hypoxia inhibits the synthesis of arginine from citrulline (Su and Block 1995).

### Changes in lipid catabolism in DS

Fatty acid oxidation is the mitochondrial aerobic process of breaking down fatty acids into acetyl-CoA units. This process is influenced by the impaired mitochondrial oxidative phosphorylation in DS. The molecule carnitine transports long-chain fatty acids into mitochondria Carnitine exists in humans as free (unesterified) carnitine, and esterified carnitine (acyl-carnitines). Free carnitine levels were reduced in young DS children (Seven et al. 2001). Similarly, free carnitine levels were lower in the hypoxic–ischemic neonates. (Çam et al. 2005). Moreover, hypoxic–ischemic neonates had significantly increased C4, C5, C5:1, C6, C6-OH, C8 levels and decreased long-chain acylcarnitine levels (López-Suárez et al. 2019). Powers and colleagues (Powers et al. 2019) also found that C5 and C5:1 levels were significantly increased in DS.

## Potential mechanisms responsible for the development of pseudohypoxia in DS. Potential therapeutic interventions

In order to conceive potential therapeutic interventions for the DS-associated pseudohypoxia, we need to understand (or at least have some reasonable working hypotheses for) the underlying pathobiochemical mechanisms. Although to identify the most relevant pathways in a system where over a thousand genes are dysregulated may seems like looking for the “needle in the haystack”, a number of potential contributing biochemical mechanisms have been identified over the last two decades.

### Antioxidant strategies

ROS accumulation promotes oxidative modification of important biomolecules, including lipids, proteins, nucleic acids and mtDNA, altering the function of several organs or the whole organism. Although chromosome 21 also encodes superoxide dismutase (SOD), which would predict that DS cells may have an improved antioxidative potential, paradoxically ROS-related processes have been suggested to play a significant role in the development of various DS pathologies (Lott et al. 2006). The most likely explanation for this paradox may be that the increased SOD cannot fully cope with the massively increased ROS formation in DS. ROS can be produced by various sources in the cell; for instance, if the mitochondrial electron transport chain is blocked, electrons can back up and leak off from the mitochondria. Moreover, hypoxic cells have a number of additional sources of ROS, such as NADPH oxidase or xanthine oxidase. ROS is not only a product of mitochondria, but also a causative factor in mitochondrial dysfunction, which, in turn, further impairs the ability of the cell to perform oxidative phosphorylation and ATP generation (Vacca et al. 2019). Moreover, the accumulation of oxidative DNA damage in DS is considered as an early event promoting cell dysfunction of various types (including neurodegeneration and Alzheimer-like dementia, a distinctive and severe hallmark of DS) (Contestabile et al. 2010).

Although ROS can serve both beneficial and detrimental effects in various conditions, a generic therapeutic approach to improve mitochondrial function and to boost cellular bioenergetics would be to scavenge/neutralize excess ROS generation, with the expectation that such intervention may not only restore mitochondrial function but also improve DNA integrity. One of the practical limitations of antioxidant strategies is that the clinically available antioxidants have a relatively slow reaction rate with many oxidant species, and they are not catalytic (i.e. they are consumed in the reaction). This requires chronic administration, often at high doses. Several clinical trials based on antioxidant administration have been undertaken, although with poor or discordant outcomes. Administration of alpha-tocopherol, ascorbic acid, and alpha-lipoic acid over 2 years showed neither an improvement in cognitive functioning nor a stabilization of cognitive decline in over-50 years old DS individuals (Lott et al. 2011). A randomized study on 7 months old children, orally supplemented with a mixture of antioxidants (selenium, zinc, vitamin A, vitamin E and vitamin C) and/or folic acids for 18 months resulted in no change in oxidative stress nor in any improvement of psychomotor and language development (Ellis et al. 2008). A possible explanation to the ineffective antioxidant supplementation is that differences between normal and DS fetuses are already present at 11 weeks gestation, which implies that and at birth, any ROS-dependent damage has already occurred (Reynolds 2008). Although some promising results on learning and memory were obtained in the DS mouse model Ts65Dn after administration of antioxidants, such as vitamin E, clinical trial in DS individuals older than 50 displayed no significant efficacy of Vitamin E in slowing cognitive decline (Sano et al. 2016).

### Coenzyme Q10

Since DS individuals display a reduction in cellular coenzyme CoQ10 content, another line of studies aimed to test the efficiency of CoQ10 supplementation. CoQ10 is a bioactive quinone synthetized by our organism, which is ubiquitously distributed in lipid environments, acting as a lipophilic antioxidant (Tiano and Busciglio 2011). In the bloodstream, CoQ10 is associated to lipoproteins, while at cellular level it is a component of plasma and intracellular membranes (Tiano and Busciglio 2011). It is also involved in mitochondrial bioenergetics, and CoQ10 intra-mitochondrial concentration determine the efficiency of OXPHOS chain. Due to its double role in supporting mitochondrial OXPHOS and counteracting oxidative DNA damage, several trials have been undertaken to test the efficiency of CoQ10 using the active form ubiquinol or the oxidized ubiquinone as antioxidant supplement for DS. To investigate the protective effect of ubiquinone on oxidation-dependent nucleic acid modifications, Tiano and colleagues conducted three experimental studies in DS children, testing the effect of 4 mg/kg/days for 6 month, 20 months and 4 years (Tiano et al. 2011, 2012; Larsen et al. 2018). Although some age-specific reduction of DNA oxidation was observed after 20 months (Tiano et al. 2012), no effect on DNA damage was observed in the long-term treatment study (Larsen et al. 2018).

Taken together, the above studies indicate that antioxidant supplementation alone does not improve clinical outcomes in DS individuals. The reasons may be related to a combination of factors. First, it is possible that ROS generation does not play as big a role in DS as presumed. Second, it is possible that some of the biological roles of ROS are beneficial and others are detrimental, and the interventions counteract both. Third, it is possible that the antioxidants, at the doses used in the trials, do not reach sufficiently high concentrations or do not react quickly or efficiently enough with the relevant ROS species. Fourth (and this applies to all therapeutic interventions discussed in the current section), it is possible that the pathophysiological positive feedforward events have progressed beyond the point of reversibility by the time the clinical interventions were started.

### *The CBS/H*_*2*_*S pathway*

Hydrogen sulfide (H_2_S) is now accepted an endogenously produced gaseous transmitter and biological regulator. Three principal enzymes, cystathionine--lyase (CSE), cystathionine-β-synthase (CBS) and 3-mercaptopyruvate sulfurtransferase (3-MST) are responsible for mammalian H_2_S biogenesis (Szabo and Papapetropoulos 2017; Zuhra et al. 2020). CBS (encoded on chromosome 21) and 3-MST (encoded on chromosome 1) are both upregulated in DS cells (Panagaki et al. 2019; Panagaki et al. 2020; reviewed in Szabo 2020); accordingly, DS cells produce high levels of CBS. Indeed, early clinical observations have demonstrated that DS individuals have elevated plasma and urinary levels of H_2_S and its metabolites (Belardinelli et al. 2001; Kamoun et al. 2003). As reviewed recently (Szabo 2020), when cellular H_2_S levels are increased beyond an optimal concentration, H_2_S produce inhibitory bioenergetic effects (in principle, via inhibition of mitochondrial Complex IV, but also through other mechanisms) (Panagaki et al. 2019, 2020; Szabo 2020; Nicholls et al. 2013).^1^ Indeed, pharmacological inhibition or genetic silencing of CBS can restore mitochondrial function and cellular proliferation in DS fibroblasts (Panagaki et al. 2019) and it can improve neurological function in DS mice (Marechal et al. 2019). Moreover, excess H_2_S in normal fibroblasts, or overexpression of CBS in otherwise normal mice, can induce cellular bioenergetic deficits or neurological deficits, respectively (Panagaki et al. 2019; Marechal et al. 2019). As reviewed recently, there are several ways to pharmacologically counteract CBS activity or the excess H_2_S production, and some of these approaches (repurposable CBS inhibitors or H_2_S scavengers) are potentially translatable into the clinical arena (Szabo 2020; Kamoun 2019), although truly ‘pharmaceutical grade’ CBS inhibitors remain to be discovered and translated into clinical testing (Zuhra et al. 2020).

#### DYRK1

Dual-specificity tyrosine-phosphorylation regulated kinase 1A (DYRK1A) is localized in the “DS critical region” of chromosome 21 (Kimura et al. 2007; Noll et al. 2009; Guo et al. 2010; Song et al. 2015). As DYRK1A is involved in tau and amyloid precursor protein phosphorylation (Kimura et al. 2007), it is a strong candidate gene for learning defects associated with Alzheimer’s disease and DS. Among others, DYRK1A also phosphorylates enzymes related to energy pathways and the methylation cycle. DYRK1A-mediated phosphorylation at the Thr^356^ residue inhibits glycogen synthase kinase activity. Glycogen synthase kinase phosphorylates and inhibits glycogen synthase and leads to a decrease in glycogen synthesis in the liver and muscles, along with increased blood glucose (Song et al. 2015).

DYRK1A phosphorylates and activates SIRT1, an enzyme that deacetylates proteins. SIRT1 deacetylates and activates PGC-1α transcription factor leading to mitochondrial biogenesis (Guo et al. 2010). DYRK1A overexpression also reduces homocysteine levels independently of CBS expression. It increases the *S*-adenosyl-l-homocysteine hydrolase activity in the reverse direction leading to a diminution of homocysteine (Nicholls et al. 2013). Further studies are needed to clarify whether these alterations are involved in learning deficiency observed in DS or other targets of DYRK1A are more critical. Nevertheless, it was found that pharmacological inhibition of brain DYRK1A can correct recognition memory deficits in three DS mouse models, all expressing an extra copy of Dyrk1a (Nguyen et al. 2018; Kim et al. 2016). Clinical trials in adults treated with epigallocatechin-3-gallate, a natural DYRK1a inhibitor from green tea, demonstrated a modest, but detectable gain of cognition in DS individuals (de la Torre et al. 2014, 2016; Xicota et al. 2020).

### Caloric restriction

The prevalence of obesity among individuals with DS is relatively high compared to the general population (Bronks and Parker 1985; Rubin et al. 1998; Melville et al. 2005; Fonseca et al. 2005; González-Agüero et al. 2011; Soler and Xandri 2011; Bertapelli et al. 2016). To explain is phenomenon, several hypotheses have been proposed, but the underlying mechanisms have not yet been elucidated*.* The following factors have been proposed to increase the risk of obesity in DS: (1) Leptin is a hormone secreted by adipocytes, acting in the hypothalamus to suppress appetite. (2) High leptin levels in obese subjects, generally thought to be the outcome of increased leptin resistance. (3) Children with DS had increased leptin for percent body fat than their unaffected siblings. This difference may contribute to the increased risk of obesity in children with DS (Magge et al. 2008). Resting energy expenditure represents the amounts of calories required for a 24-h period by the body during a non-active period. Lower resting energy expenditure could partially explain the increased risk of obesity in youth with DS. Hill and colleagues found that children with DS had lower resting energy expenditure compared to siblings without DS (Hill et al. 2013; Mendonca et al. 2010). Whether traditional dietary interventions (e.g. caloric restriction) have the potential to improve metabolic status (beyond improving BMI, for instance), remains to be seen.

### Low sulfur diet

Generally, a low sulfur diet involves the reduction of meats, dairy products, eggs, onions, peas and cruciferous vegetables. The primary aim of a potential low sulfur diet would be to reduce the generation of H_2_S which is the result of increased CBS and 3-MST activity (see above). Amino acids, methionine and cysteine are the primary sources of sulfur. A mathematical model (Orozco et al. 2019) suggests that a low-methionine diet might offer a perspective for reversing the metabolic imbalance in DS, but clinical investigations remain to be conducted to test this theory.

### Vitamin supplementation (folate and cobalamine)

Folate is an important vitamin that contributes to cell division and growth. Studies on the effect of vitamin supplements on metabolic or clinical outcomes in DS showed limited, if any, evidence of improvement (Lumley et al. 2001; Blehaut et al. 2010).

### Choline supplementation

Supplementing the maternal diet of Ts65Dn mice with choline during pregnancy and lactation substantially improved attention (Strupp et al. 2016). Therefore, choline metabolism might be an important target in DS. However, there are no clinical studies on choline supplementation in DS.

### Amino acid supplementation

Lejeune et al. has found differences in serine concentrations between control children and DS individuals and recommended a dietary supplementation (Lejeune et al. 1992). Many of the currently marketed food supplement formulas for DS include amino acids.

### Zinc and selenium

Several studies have shown zinc and selenium serum levels are decreased in DS (Barañano and Hartman 2008). These trace elements are involved in the synthesis and regulation of the activity of proteins and enzymes that are necessary for our endogenous antioxidant response (Saghazadeh et al. 2017; Parisotto et al. 2015). It has been suggested that antioxidant therapies might have some benefit for DS individuals, although the clinical trials have not been particularly encouraging (see above).

### Ketogenic diet

The ketogenic diet contains high fat, moderate protein, and very low carbohydrate, which induce the hepatic production of ketone bodies. Ketone bodies cross the blood–brain barrier and are converted to acetyl coenzyme A, enters the citric acid cycle to produce energy for the brain. The ketogenic diet traditionally has been used in cases of intractable epilepsy (Barañano and Hartman 2008). As epilepsy is a common comorbidity in DS and it is also thought to contribute to cognitive dysfunction for cognitive decline in DS, it was proposed that the ketogenic diet may be a therapeutic option for cognitive decline in DS (Kaneko et al. 2018).

### Physical exercise

Lower physical activity due to overall weakness has been well documented in DS (Cioni et al. 1994; Croce et al. 1996; Carmeli et al. 2002; Hawli et al. 2009; Bala et al. 2018; Dupre and Weidman-Evans 2017; Coelho-Junior et al. 2019; Aguiar et al. 2008; Pastore et al. 2000; Phillips et al. 2013). Consumption of high carbohydrate and nutrient-poor food products is more frequent in DS children as these products are easy to chew. The increased expression and activity of CBS in children with DS results in an increased plasma level of cysteine (Pogribna et al. 2001). Plasma concentrations of cysteine correlate strongly with fat mass, but the mechanism of how increased cysteine levels induce fat accumulation is unclear (Elshorbagy et al. 2012). One can also postulate that beta-oxidation of fatty acids in DS is impaired as the mitochondrial electron transport chain is not functioning properly, producing increased acyl-carnitine levels.

Physical exercise may improve DS outcomes through effects on the mitochondria. It is known that electron transport chain activity i.e. mitochondrial function, and mitochondrial numbers can improve with exercise training (Menshikova et al. 2006). Mitochondrial function is regulated by mitochondrial matrix free Ca^2+^ concentration. Stimuli such as action potentials or hormone-receptor bindings can trigger a signal transduction process leading to Ca^2+^ oscillations between intracellular organelles (Pecze et al. 2015). This leads to the activation of mitochondrial dehydrogenases (Denton 2009). Moreover, mitochondria undergo several structural changes in response to the energetic challenge. High energy demand induces mitochondrial fusion allowing efficient mixing of mitochondrial content within an extended mitochondrial network (Westermann 2012). Activation of the PGC-1*α* transcription factor, the “master regulator” of mitochondrial biogenesis is also triggered by physical exercise (Drake et al. 2016). PGC-1*α* regulates the expression of nuclear genes encoding mitochondrial proteins, thereby regulating mitochondrial content and function (Hood et al. 2016). All of the abovementioned changes would be expected to exert beneficial effects in DS individuals. Functional fitness in DS can be meaningfully improved through physical exercise (Dodd and Shields 2005). For instance, a non-randomized controlled trial has found that the functional fitness of adults with DS improved with an aquatic training but was insufficient to improve balance and upper body strength (Boer and de Beer 2019).

## Implications, conclusions and outlook

Since mitochondrial function is the crucial contributor to ATP generation in mammalian cells, and ATP is essential to the maintenance of all fundamental cellular function in resting cells, (and even more so in dividing ones), one would expect that a significant impairment of cellular ATP synthesis would result in significant suppression of all fundamental cellular functions, including cellular division cell proliferation, maintenance of membrane potential, and—for neurons—cellular excitability ability to form synapses, release neurotransmitters and induce post-synaptic currents. Indeed, multiple studies demonstrate that DS cells have an impairment in all of the above-mentioned processes, and there is evidence that various interventions aimed at restoring mitochondrial activity can improve these functional parameters (Izzo et al. 2017; Panagaki et al. 2019; Park et al. 2000; Jablonska et al. 2006; Contestabile et al. 2007; Trazzi et al. 2011; Valenti et al. 2013; Weick et al. 2013; Bhattacharyya et al. 2009; Stern et al. 2015; Coskun et al. 2017; Huo et al. 2018; Mizuno et al. 2018; Mollo et al. 2019).

A generalized energetic deficit would also predict disruptions in the cell cycle and impairment of nuclear and mitochondrial DNA repair (all extremely energetically demanding processes), contributing to genomic instability. Indeed, there is direct experimental evidence showing significant impairment of DNA repair and cell cycle in human DS cells and in animal models of DS (e.g. Contestabile et al. 2007; Pincheira et al. 1994; Druzhyna et al. 1998; Maluf and Erdtmann 2001; Rosner et al. 2003; Necchi et al. 2015; Wang et al. 2016). While various compensatory bioenergetic processes (e.g. an upregulation of glycolysis) may be able to ‘make up for’ some of the ‘missing’ ATP), the consequences of the metabolic pseudohypoxia may be more severe when the cells experience sudden increases in energetic demand. For instance, it is conceivable that such fundamental bioenergetic mechanisms may contribute to the well-documented impairment of exercise tolerance in DS individuals (see: Introduction). An impaired mitochondrial function in DS may also predict that DS tissues have a reduced O_2_ utilization; for example, measured as whole-body-O_2_ consumption, or as tissue O_2_ extraction (i.e. quantification of arteriovenous oxygen difference). Such measurements are commonplace in the field of metabolic disease and in the field of sepsis (where, in many pathophysiological conditions, the tissues’ ability to extract O_2_ is impaired; for instance, in sepsis, due to a metabolic ‘poisoning’ of the mitochondria) (Carré and Singer 2008); indeed, the available body of clinical data in DS indicates that a similar reduced O_2_ utilization is characteristic of DS individuals (Seron and Greguol 2014; Mendonca et al. 2018). The same process (impaired mitochondrial function, and the resulting compensatory upregulation of glycolysis) would also produce increases in systemic lactate production (again, a common observation in septic shock, where, in fact, elevated lactate levels are excellent predictors of mortality); indeed, our meta-analysis has clearly demonstrated that DS cells produce increased levels of lactate (Fig. 4c) and clinical studies have also reported elevated plasma lactate levels in DS individuals (Caracausi et al. 2018; Gross et al. 2019).

It is plain to see how a generalized cellular bioenergetic deficit (pseudohypoxia) would predispose to various embryonic developmental problems and to various fundamental functional disturbances in later life. These problems would be expected even on the background of a (otherwise unperturbed) organism (i.e. in the absence of any additional gene dysregulation); it is obvious that a globalized bioenergetic deficit would be even more deleterious in cells where hundreds of genes and gene products are dysregulated due to the gene dosage effect of chromosome 21, as well as the even larger number of perturbances of genes encoded on all other chromosomes. Let’s consider one single bioenergetic disturbance, the inhibition of mitochondrial Complex IV activity (which is reversibly inhibited by H_2_S and irreversibly inhibited by cyanide). It is well documented in the environmental toxicology literature that even a partial inhibition of this enzyme (caused by H_2_S or cyanide administration) can cause various developmental and neurodevelopmental defects in developing embryos (Singh 1981; Tewe and Maner 1981; Frakes et al. 1986; Hannah and Roth 1991; Skrajny et al. 1992; U.S. EPA 2010); Complex IV inhibition also produces significant bioenergetic and functional (including neurocognitive) defects in juvenile and adult organisms (Egekeze and Oehme 1980; Persson et al. 1985; Nicholson et al. 1998; Schneider et al. 1998; Nam et al. 2004; Fiedler et al. 2008; Reed et al. 2014; Tshala-Katumbay et al. 2016). In conditions with insufficient energy production in the brain, convulsions also frequently occur (Folbergrovà et al. 1981; Nakanishi et al. 1991; Shukla et al. 1989). Indeed, there is a significant increase in the incidence of convulsive events in DS individuals (Bull et al. 2011; Barca et al. 2014).

A global pseudohypoxia, of course, would be also expected to be a proximate cause of neurological dysfunction (since neurons are highly ATP-dependent cells, highly susceptible to disturbances in cellular bioenergetic processes) (Hoyer 1990; Zeiger et al. 2011; Funes et al. 2014; Nicholls et al. 2015; Hebert-Chatelain et al. 2016; Plotegher et al. 2020). We hypothesize that neuronal pseudohypoxia may also potentially contribute to the process of delayed neurodegeneration (Alzheimer-like symptoms) in DS. Indeed, DS cells exhibit significant problems with protein processing (Aivazidis et al. 2017), and it is well documented that cellular bioenergetic deficit, on its own, can directly causes problems with the processing of various cellular proteins, including amyloid beta (Hoyer 1993; Gabuzda et al. 1994; Meier-Ruge et al. 1994; Busciglio et al. 2002; Ferreira et al. 2010; Wilkins and Swerdlow 2017). It is conceivable that the chronic cellular bioenergetic deficit in DS contributes to dysfunction amyloid processing, which, in turn, may culminate in an Alzheimer’s-like neurodegenerative process. This hypothesis, nevertheless, remain to be further investigated in future studies.

In the current analysis, we have primarily focused on studies utilizing *human* DS cells or on clinical studies on DS individuals (as opposed to animal studies). There are, in fact, multiple DS mouse models available (reviewed in Gupta et al. 2016; Herault et al. 2017; Muñiz Moreno et al. 2020), and these models do mimic some of the features of the human DS. However, in mice, the genes that are on human chromosome 21 are localized on 3 different chromosomes, and in most published studies utilizing various DS mice strains use animal models that do not overexpress all of the human-DS genes. Therefore, in some case these models are useful and potentially clinically translatable, while in others they may not be. For instance, one of the most commonly used mouse model (Ts65Dn) overexpresses DYRK1, and therefore it may be considered an adequate model of the human DS (at least as far as DYRK1-related processes are concerned). However, the same mouse not overexpress the mouse-equivalent of the human *cbs* gene; given the emerging evidence for the potential role of CBS and H_2_S in DS, the utility of this particular mouse strain to study H_2_S-related processes is questionable. Clearly, future animal studies of DS should utilize mice where the set of ‘gene dosage’ closely approximates the human condition. While such animals (containing segments of all relevant 3 extra mouse chromosomes) have been created, they are fragile and have significant fertility problems, limiting their utility for experimentation (Yu et al. 2010). There are also mouse models overexpressing the human chromosome 21, or part of it (O’Doherty et al. 2005; Miyamoto et al. 2014; Kazuki et al. 2020); while these animals have the ‘correct’ extra genes, these are human genes encoding human proteins: the interaction of these human proteins with the mouse proteins is likely to be different than human–human or mouse-mouse protein–protein interactions, which introduces a significant complicating factor. Clearly, the field of DS research requires better animal tools, and undoubtedly, such tools will be forthcoming in the future.

The radical therapy of DS, i.e. complete switching off the extra chromosome in all somatic cells is, of course, the logical “definitive therapeutic solution”. (Although, even in this case, one would have to consider what would be the ideal timing of such intervention, perhaps intrauterine, because by the time of birth, significant, perhaps irreversible developmental problems can occur). Such studies have been pursued by several laboratories and turning off chromosome 21 has been successful in cell-based models (Jiang et al. 2013; Mentis 2016; Chiang et al. 2018). However, it remains unclear if/when these technical advances (sometimes known as ‘chromosome therapy’) would be clinically translatable. In the meantime, continuing experimental studies, both omics-based and hypothesis-driven, are expected to further elucidate already identified targets, identify potentially translatable pharmacological or nutritional tools to target them, and to identify novel targets for pharmacological interventions in DS.

The (generally) disappointing experience with clinical trials in DS, so far, can be attributed to many different factors (Gardiner 2014; Lee et al. 2020a, b), some of these issues relate to clinical trial design (duration, dosage), while others may relate to the fact that possibly in the trials so far, the pathways/targets were not the most relevant for the pathogenesis of DS. Other reasons may include the relative ‘bluntness’ of the pharmacological tools tested so far (for instance, insufficient potency/specificity and/or suboptimal dose of the antioxidants used in the trials). From the pathomechanisms and associated therapeutic interventions discussed in “Changes in lipid catabolism in DS” section, in our opinion, for the therapeutic improvement of the pseudohypoxic state in DS, ROS scavengers/antioxidants (provided that agents of sufficient activity, perhaps catalytic antioxidants) may be worth for renewed attention. Moreover, future generations of DYRK1 inhibitors and/or CBS inhibitors may be promising approaches as they appear to target important checkpoints in the pathophysiology of DS. It is also conceivable that a complex condition such as DS requires simultaneous pharmacological intervention at multiple ‘levels’, similar to what is considered common practice, for instance, in cancer therapy. These issues remain to be tackled both at the level of future experimental and preclinical studies as well as during the next wave of clinical trials.

## Conclusion

**Fig. 6:**
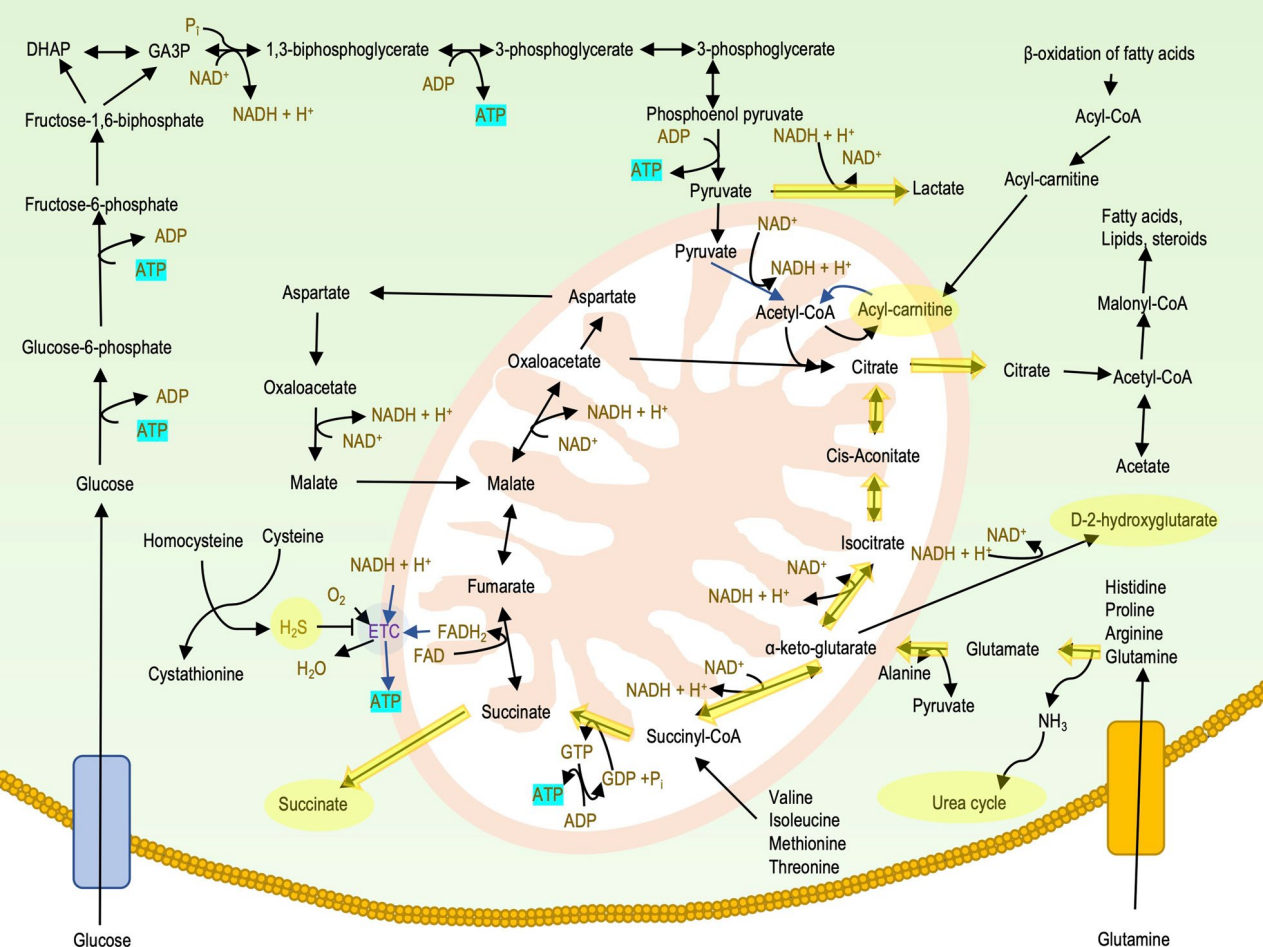
Metabolic and bioenergetic alterations in DS. Upregulated pathways and metabolites are highlighted in yellow. As mitochondrial oxidative phosphorylation is suppressed in DS, cells downregulate various proteins of the electron transport chain and re-wire their energetic pathways. Glycolysis is upregulated, producing ATP and as a byproduct, lactate. Because the mitochondrial NADH consumption is inhibited, NADH does not enter the mitochondria through the malate-aspartate cycle, but, rather, it is converted back to NAD^+^ by lactic fermentation. As pyruvate is transformed into lactate, it does not enter the cyclic acid cycle. Similarly, lipids are also inhibited to enter the Krebs cycle and they are accumulated in the form of acyl-carnitines. As another compensatory pathway, glutamine, glutamate and other amino acids enter the Krebs cycle as 2-oxoglutarate and generate GTP, which can immediately be transformed to ATP and NADH. To oxidize the NADH, the Krebs cycle can run in reverse direction, thus producing citrate. As a result, various metabolites and enzymes involved in cyclic acid cycle are upregulated. As cells use amino acids as an alternative energy source, excess ammonia is generated (this can, in turn, be eliminated via the urea cycle)

The metabolomic alterations, as shown in the above sections, as well as in the Additional file 1/Supplement, as summarized in Fig. 6, suggest that DS cells are in a pseudo-hypoxic state: they show pathophysiological alterations that resemble cellular responses to hypoxia, even though their oxygen supply is not disrupted. This alteration may importantly contribute a variety of functional deficits associated with DS. Several potential therapeutic approaches exist to counteract this DS-associated pseudohypoxia.

## Supplementary information

**Additional file 1**. Supplementary material. ** Supplementary Fig. 1**. Log2 transformation and normalization on raw datasets. The boxplot shows the distribution of plasma samples after log2 transformation. Data are from Caracausi et al. 2018. **Supplementary Fig. 2**. Log2 transformation and normalization on raw datasets. The boxplot shows the distribution of plasma samples after log2 transformation. Data are from Antonaros et al. 2020. **Supplementary Fig. 3**. Boxplot showing the distribution of plasma samples after log2 transformation. The authors (Caracausi et al. 2018) used Probabilistic Quotient Normalization (PQN). **Supplementary Fig. 4**. Boxplot showing the distribution of plasma samples after log2 transformation. Normalized and log2 transformed samples from the study of Powers et al. 2019. **Supplementary Fig. 5**. Targeted metabolomic result from Culp-Hill study (Culp-Hill et al. 2017). **Supplementary Fig. 6**. Untargeted metabolomic result from Culp-Hill study (Culp-Hill et al. 2017) showing significant technical variations. **Supplementary Fig. 7**. Untargeted metabolomic result from Culp-Hill study Culp-Hill et al. 2017). We applied variance stabilization normalization to reduce technical variations. **Supplementary Table 1**. Significant regression coefficients from a multiple linear regression model applied on each metabolite found in plasma. The raw data are from Caracausi’s study (Caracausi et al. 2018). **Supplementary Table 2**. Significant regression coefficients from a multiple linear regression model applied on each metabolite found in urine. The raw data are from Caracausi’s study (Caracausi et al. 2018). **Supplementary Fig. 8**. Energy charge values in DS cells vs. control. **Supplementary Fig. 9**. Meta-analysis of amino acids not included in the Main Text.

## Data Availability

Not applicable.

## Abbreviations

ATP: Adenosine-5′-triphosphate
ADP: Adenosine-5′-diphosphate
AMP: Adenosine-5′-monophosphate
2,3-DPG: 2,3-Diphosphoglyceric acid
GTP: Guanosine-5′-triphosphate
NADH: Reduced nicotinamide adenine dinucleotide
FADH_2_: Reduced flavin adenine dinucleotide
NADPH: Reduced nicotinamide adenine dinucleotide phosphate
CoA: Coenzyme A
DS: Down syndrome
DYRK1A: Dual-specificity tyrosine-phosphorylation regulated kinase 1A
M: Mean
Pi: Inorganic phosphate
SD: Standard deviation
SEM: Standard error of mean N sample size
sqrt: Square root of
ROS: Reactive oxygen species
CBS: Cystathionine beta-synthase

## Footnotes

1 Technical footnote: with a few recent exceptions (Panagaki et al. 2019; Panagaki et al. 2020), most of the studies investigating mitochondrial function, or specific function of various mitochondrial complexes in DS utilized isolated mitochondrial preparations (in many cases also using high saturating concentrations of electron donors). These experiments would ‘miss’ the inhibitory effect of a gaseous mediator, such as H_2_S, which would no longer be present in the mitochondria after subcellular isolation and in vitro processing. Moreover, none of the studies focusing on the expression of mitochondrial electron complexes (e.g. Table 2) would “see” a H_2_S-mediated inhibition of Complex IV, because this occurs on the enzymatic activity level, and not on the level of protein expression. And yet, such an inhibitory effect can markedly suppress the activity of the entire mitochondrial electron transport, and, in our opinion, is therefore more functionally relevant than the expression (omics) type surveys.
