# Supplementary material for: Meta-analysis of metabolites involved in bioenergetic pathways reveals a pseudohypoxic state in Down syndrome
Source: Mol Med. 2020 Nov 9;26:102. doi: 10.1186/s10020-020-00225-8 (PMC7653803; doi:10.1186/s10020-020-00225-8)
Supplement: Supplementary file 1 — Additional file 1. Supplementary material. Supplementary Fig. 1. Log2 transformation and normalization on raw datasets. The boxplot shows the distribution of plasma samples after log2 transformation. Data are from Caracausi et al. 2018. Supplementary Fig. 2. Log2 transformation and normalization on raw datasets. The boxplot shows the distribution of plasma samples after log2 transformation. Data are from Antonaros et al. 2020. Supplementary Fig. 3. Boxplot showing the distribution of plasma samples after log2 transformation. The authors (Caracausi et al. 2018) used Probabilistic Quotient Normalization (PQN). Supplementary Fig. 4. Boxplot showing the distribution of plasma samples after log2 transformation. Normalized and log2 transformed samples from the study of Powers et al. 2019. Supplementary Fig. 5. Targeted metabolomic result from Culp-Hill study (Culp-Hill et al. 2017). Supplementary Fig. 6. Untargeted metabolomic result from Culp-Hill study (Culp-Hill et al. 2017) showing significant technical variations. Supplementary Fig. 7. Untargeted metabolomic result from Culp-Hill study Culp-Hill et al. 2017). We applied variance stabilization normalization to reduce technical variations. Supplementary Table 1. Significant regression coefficients from a multiple linear regression model applied on each metabolite found in plasma. The raw data are from Caracausi’s study (Caracausi et al. 2018). Supplementary Table 2. Significant regression coefficients from a multiple linear regression model applied on each metabolite found in urine. The raw data are from Caracausi’s study (Caracausi et al. 2018). Supplementary Fig. 8. Energy charge values in DS cells vs. control. Supplementary Fig. 9. Meta-analysis of amino acids not included in the Main Text. [file 10020_2020_225_MOESM1_ESM.pdf]

<u>Supplementary material</u>

Main Text: "Meta-analysis on metabolites involved in bioenergetic pathways reveals a pseudohypoxic state in Down syndrome"

Laszlo Pecze, Elisa B. Randi and Csaba Szabo

1. Log2 transformation and normalization on raw datasets

Caracausi et al. (2018) and Antonaros et al. (2020) used the unnormalized raw data for the evaluation of metabolic profile in plasma from DS and control children. Although there are no significant variations between samples (Supplementary Figs. 1-2), it cannot be excluded that a normalization procedure would have a beneficial effect. Nevertheless, we left it unnormalized for our meta-analysis. Caracausi et al. also reported metabolite levels in urine from DS and control children. They normalized the raw data according to PQN method (Dieterle et al., 2006). After normalization data show only small variability between samples (Supplementary Fig. 3).

Powers et al. (2019) used the removeBatchEffect function from R limma package (Ritchie et al., 2015) to normalize the raw data. The result of the normalization is shown in Supplementary Fig.4.

Culp-Hill et al. (2017) provided two datasets one is from targeted experiment and one from untargeted one. The targeted dataset does not show significant variation between samples. See Supplementary Fig. 5. However, there are significant variations between samples on the nontargeted metabolic result. See Supplementary Fig 6. Using a Wilcoxon non-parametric test on the average of the samples we have found that there is a significant difference between DS and control. (p-value < 0.0001). This means that, if the data are used without normalization, each component will show significantly higher values for DS group. Unfortunately, this is the case in the published article. Therefore, we used variance stabilization normalization (Huber et al., 2002) to implement these data into our meta-analysis. (Supplementary Fig. 7).

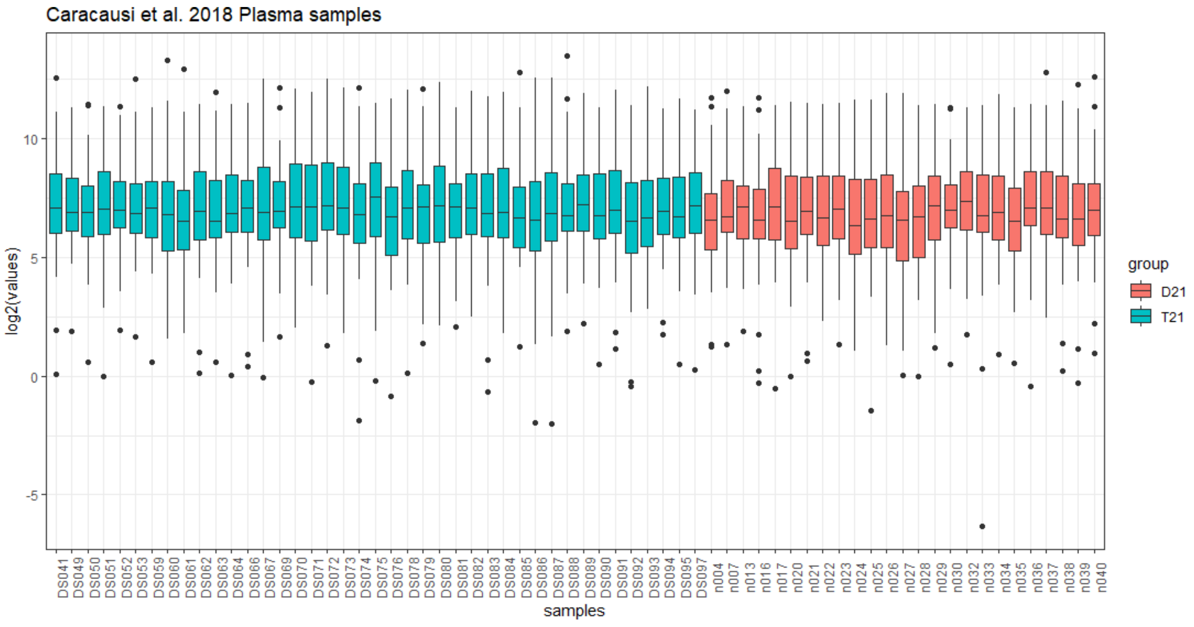

Supplementary Fig.1. Log2 transformation and normalization on raw datasets. The boxplot shows the distribution of plasma samples after log2 transformation. Data are from Caracausi et al., 2018.

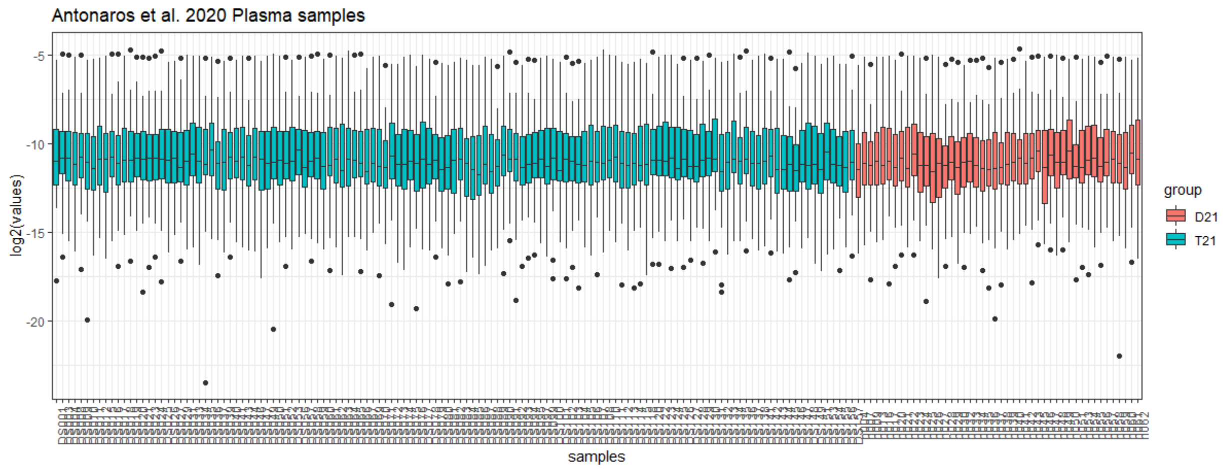

Supplementary Fig.2. Log2 transformation and normalization on raw datasets. The boxplot shows the distribution of plasma samples after log2 transformation. Data are from Antonaros et al., 2020.

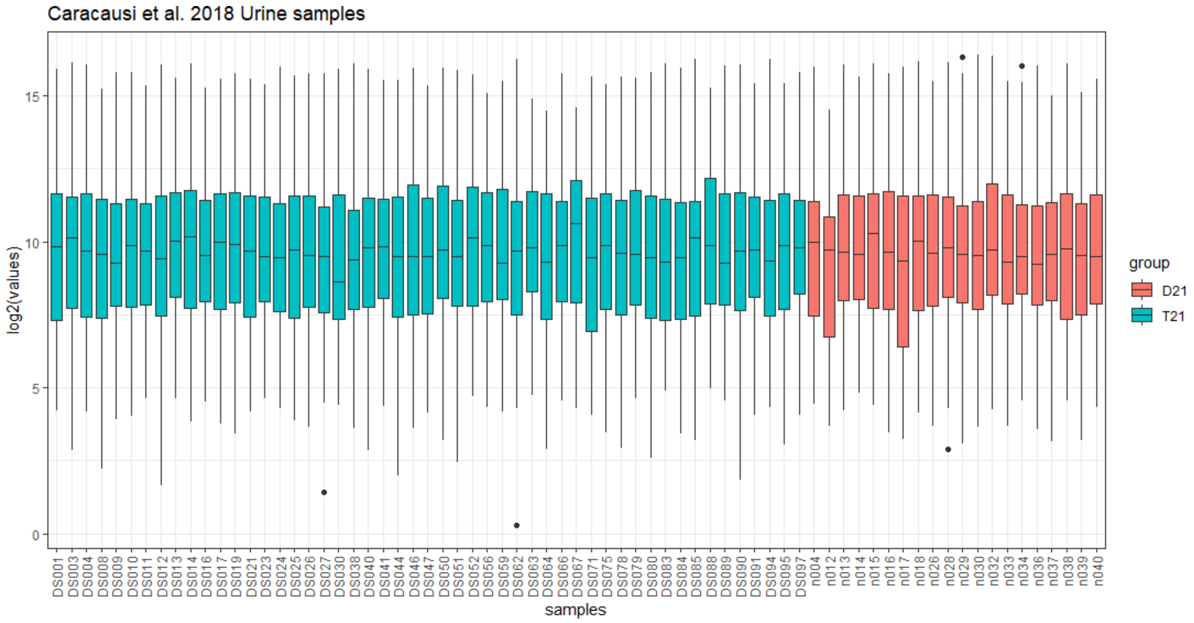

Supplementary Fig. 3. Boxplot showing the distribution of plasma samples after log2 transformation. The authors (Caracausi et al., 2018) used Probabilistic Quotient Normalization (PQN).

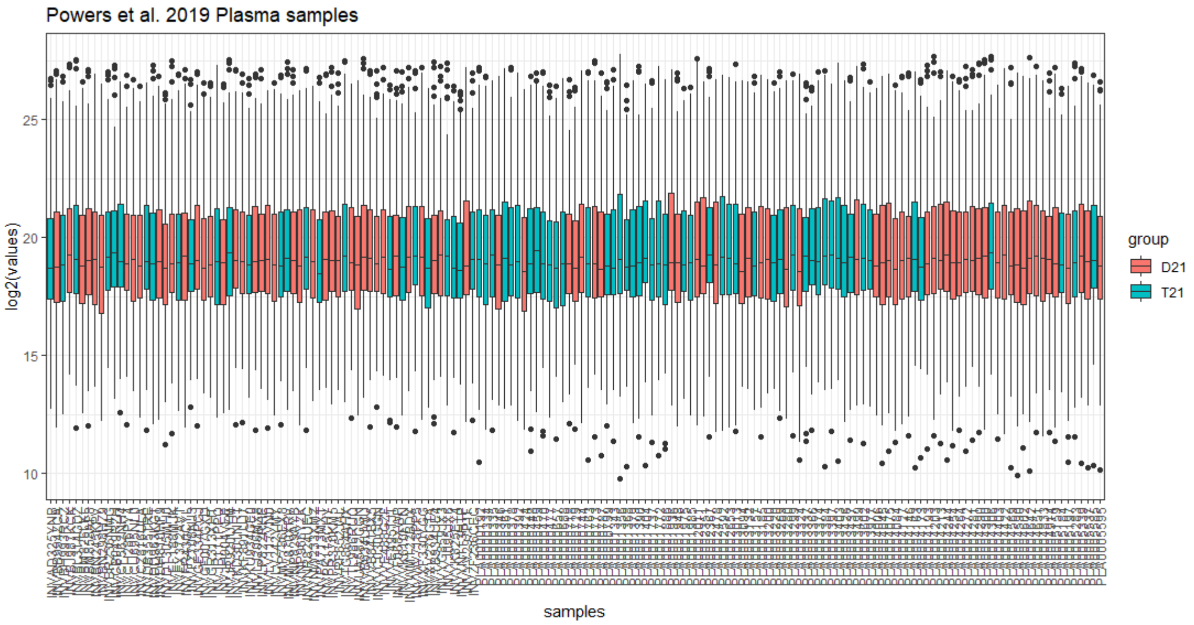

Supplementary Fig.4. Boxplot showing the distribution of plasma samples after log2 transformation. Normalized and log2 transformed samples from the study of Powers et al., 2019.

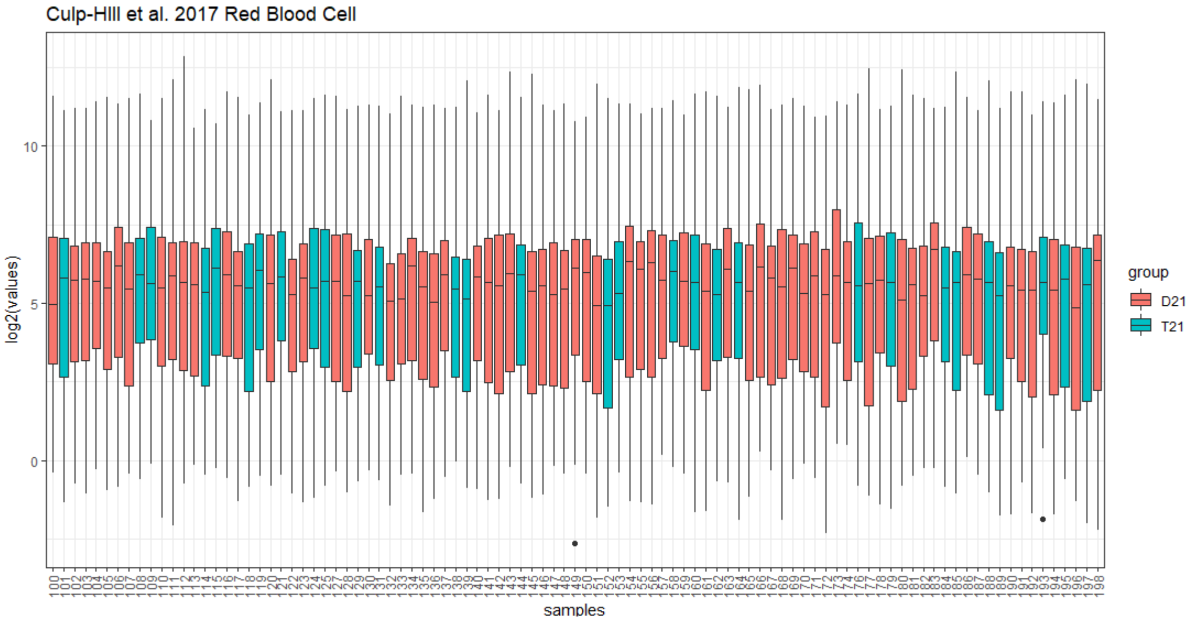

Supplementary Fig. 5. Targeted metabolomic result from Culp-Hill study (Culp-Hill et al., 2017).

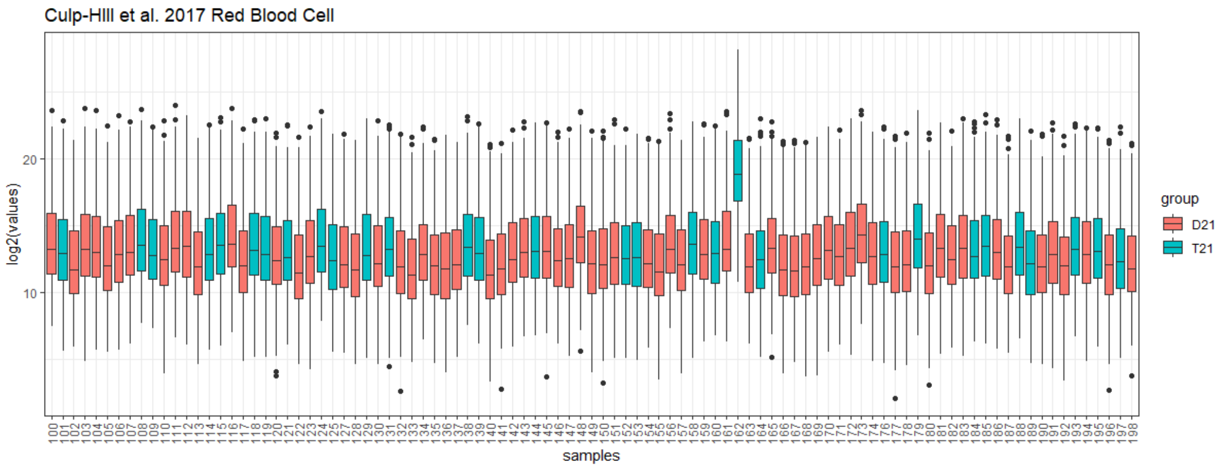

Supplementary Fig. 6. Untargeted metabolomic result from Culp-Hill study (Culp-Hill et al., 2017) showing significant technical variations.

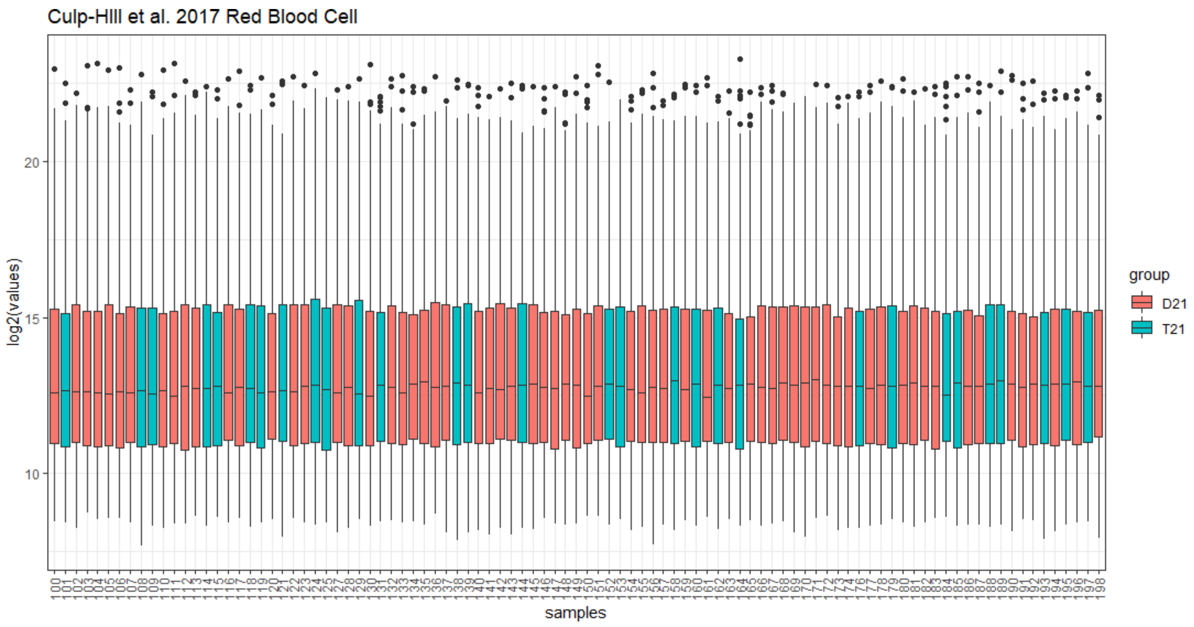

Supplementary Fig.7. Untargeted metabolomic result from Culp-Hill study (Culp-Hill et al., 2017). We applied variance stabilization normalization to reduce technical variations.

2. Impact of fasting state on metabolic levels

Caracausi and colleagues (Caracausi et al., 2018) provided information on the fasting state of subjects i.e breakfast has been taken before sample collection or not. In order to know the impact of fasting on the levels of metabolites in plasma, we performed a multivariate linear regression, where the amount of a given metabolite is the dependent variable while Karyotypes (T21/D21), Gender (Male/Female), Age and Fasting (Yes/No) are the independent variable. If the regression coefficient statistically differs from zero, i.e. there is an effect, the regression coefficient was put into a table with its 95% confidence intervals. The result is shown in the Supplementary Table 1. We can conclude that fasting has an impact on the levels of glucose and the ketone bodies, but it has no significant effect on other metabolites. We also checked the impact of fasting on metabolite levels using the same multivariate regression model. Data are shown on Supplementary Table 2. In general, we can conclude that accounting for sex, age, and fasting state did not significantly affect the main result.

**Compound**	**KaryotypeT21**<br>β [95% CI]	**FastingYes**<br>β [95% CI]	**Age**<br>β [95% CI]	**SexM**<br>β [95% CI]
Leucine	NS	NS	0.01 [0, 0.02]	0.17 [0.01, 0.32]
Isoleucine	NS	NS	0.01 [0, 0.03]	NS
Valine	NS	NS	NS	NS
Lactate + Threonine	NS	NS	-0.02 [-0.04, 0]	NS
Alanine	NS	NS	NS	NS
Acetate	0.56 [0.15, 0.96]	NS	NS	NS
Pyruvate	0.24 [0.04, 0.44]	NS	-0.02 [-0.03, -0.01]	NS
Acetone	0.5 [0.08, 0.92]	0.61 [0.14, 1.08]	NS	NS
Glutamine	NS	NS	NS	NS
Citrate	NS	NS	NS	NS
Glycine	NS	NS	NS	NS
Creatine	NS	NS	-0.05 [-0.08, -0.02]	NS
Creatinine	NS	NS	0.06 [0.04, 0.07]	NS
Lactate	NS	NS	-0.02 [-0.04, 0]	NS
Glucose	NS	-0.1 [-0.2, -0.01]	NS	NS
Mannose	NS	NS	NS	NS
Tyrosine	-0.21 [-0.41, 0]	NS	NS	NS
Histidine	NS	NS	NS	NS
Phenylalanine	NS	NS	NS	NS
Formate	0.47 [0.18, 0.76]	NS	NS	NS
Fumarate	NS	NS	NS	NS
Threonine	NS	NS	NS	NS
Glycerol	0.9 [0.19, 1.62]	NS	NS	NS
Lysine	NS	NS	NS	NS
Acetatoacetate	NS	NS	NS	NS
Methionine	NS	NS	NS	NS
Unk1	NS	0.28 [0.05, 0.51]	NS	NS
Unk2	NS	NS	NS	NS
Unk3	NS	NS	NS	NS
3-hydroxybutyrate	NS	0.87 [0.15, 1.58]	-0.05 [-0.09, -0.01]	NS
Succinate	0.6 [0.34, 0.86]	NS	NS	NS
2-hydroxybutyrate	NS	NS	NS	NS
Glutamate	NS	NS	-0.08 [-0.14, -0.01]	NS

Supplementary Table 1. Significant regression coefficients from a multiple linear regression model applied on each metabolite found in plasma. The raw data are from Caracausi's study (Caracausi et al., 2018).

**Compound**	**KaryotypeT21**<br>β [95% CI]	**Fasting Yes**<br>β [95% CI]	**Age**<br>β [95% CI]	**SexM**<br>β [95% CI]
Fumarate	NS	NS	-0.01 [-0.03, 0]	NS
DMG	NS	NS	NS	NS
Allantoin	NS	NS	NS	NS
Acetone	0.45 [0.07, 0.82]	NS	NS	NS
Isoleucine	NS	NS	NS	NS
Leucine	NS	NS	NS	NS
Valine	NS	NS	NS	NS
Alanine	NS	NS	NS	NS
Asparagine	NS	NS	NS	NS
Creatine + Creatinine	NS	NS	0.04 [0.02, 0.05]	NS
Citrate	NS	NS	NS	-0.59 [-1.03, -0.15]
Glycine	-0.48 [-0.94, -0.02]	NS	NS	NS
Ethanolamine	NS	NS	0.01 [0, 0.03]	NS
4-hydroxyphenylacetate	NS	NS	NS	NS
3-hydroxyisovalericacid	NS	NS	NS	NS
Dimethylamine	NS	NS	NS	NS
Trimethylamine-N-oxide	0.79 [0.3, 1.27]	NS	NS	NS
Taurine	NS	NS	NS	NS
Creatine	NS	NS	-0.18 [-0.25, -0.1]	NS
Glycolate	NS	NS	NS	NS
Creatinine	NS	NS	0.05 [0.04, 0.07]	NS
Tyrosine	0.21 [0.01, 0.42]	NS	NS	NS
Hippurate	NS	NS	NS	NS
Formate	-0.53 [-1.01, -0.04]	NS	-0.04 [-0.07, -0.01]	NS
Trigonelline	NS	NS	NS	NS
1-methylnicotinamide	NS	NS	NS	NS
Phenylacetylglycine	0.44 [0.03, 0.85]	NS	NS	NS
2-hydroxyisobutyricacid	NS	NS	NS	NS
Glutamate + Glutamine	NS	0.08 [0, 0.17]	-0.01 [-0.02, -0.01]	NS
Lysine	-0.21 [-0.38, -0.03]	NS	NS	NS

Supplementary Table 2. Significant regression coefficients from a multiple linear regression model applied on each metabolite found in urine. The raw data are from Caracausi's study (Caracausi et al., 2018).

3. **Energy charge**

The energy charge is related to ATP, ADP and AMP concentrations to account for the energy status in metabolism (Atkinson and Walton, 1967). Energy charge is defined as:

Energy charge = ([ATP] + 1/2[ADP]) / ([ATP]+[ADP]+[AMP])

The energy charge of most cells varies between 0.7 and 0.95 (Atkinson and Walton, 1967). The energy charge was calculated DS and CTR cells, if the study provides information for all the metabolites i.e. ATP, ADP, and AMP (Supplementary Fig. 8). Although the cells from Down syndrome subjects are less charged, the change did not reach the significance level at p>0.05 (using a paired t-test).

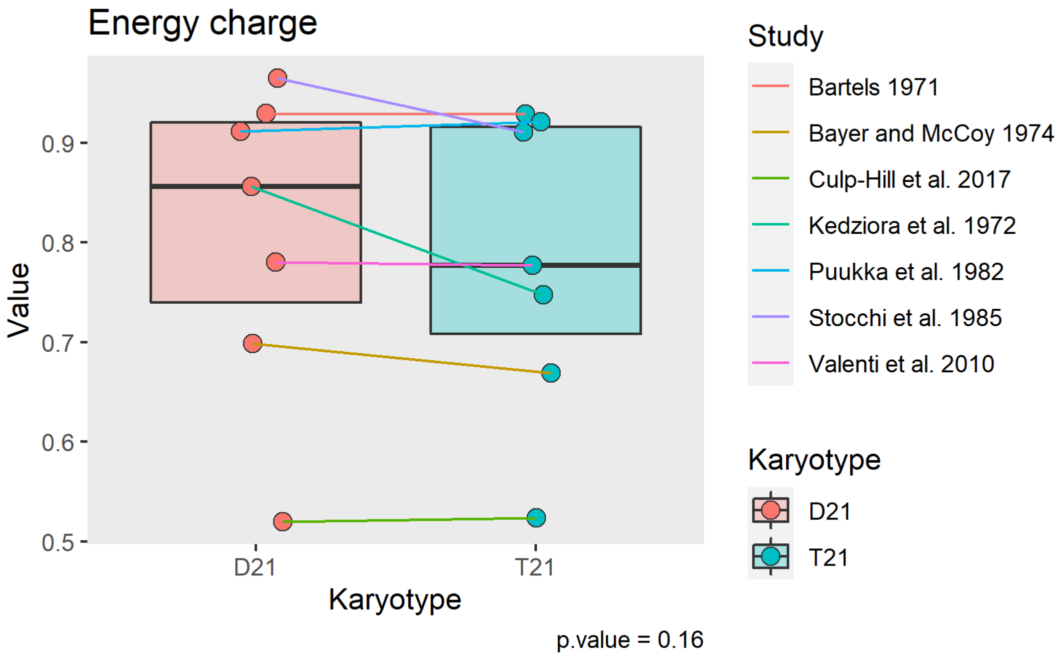

Supplementary Fig. 8. Energy charge values in DS cells vs. control.

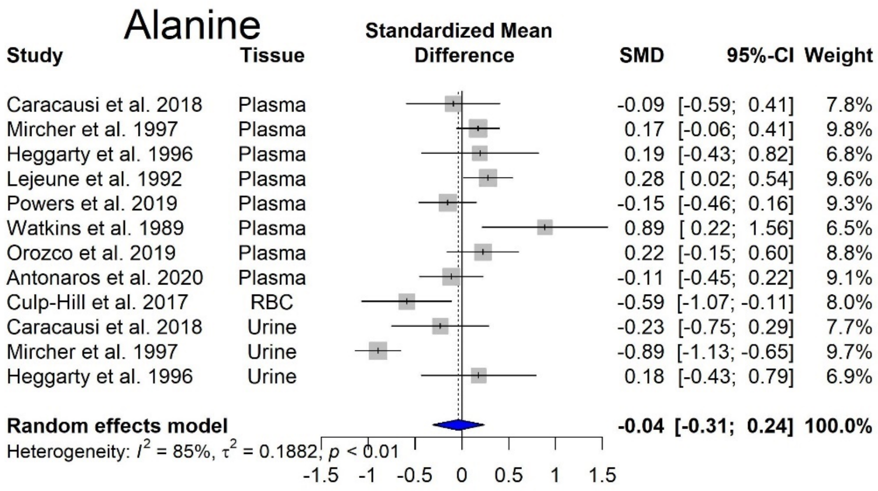

Alanine
Standardized Mean Difference
Study
Tissue
SMD
95%-CI
Weight
Caracausi et al. 2018 Plasma -0.09 [-0.59; 0.41] 7.8%
Mircher et al. 1997 Plasma 0.17 [-0.06; 0.41] 9.8%
Heggarty et al. 1996 Plasma 0.19 [-0.43; 0.82] 6.8%
Lejeune et al. 1992 Plasma 0.28 [0.02; 0.54] 9.6%
Powers et al. 2019 Plasma -0.15 [-0.46; 0.16] 9.3%
Watkins et al. 1989 Plasma 0.89 [0.22; 1.56] 6.5%
Orozco et al. 2019 Plasma 0.22 [-0.15; 0.60] 8.8%
Antonaros et al. 2020 Plasma -0.11 [-0.45; 0.22] 9.1%
Culp-Hill et al. 2017 RBC -0.59 [-1.07; -0.11] 8.0%
Caracausi et al. 2018 Urine -0.23 [-0.75; 0.29] 7.7%
Mircher et al. 1997 Urine -0.89 [-1.13; -0.65] 9.7%
Heggarty et al. 1996 Urine 0.18 [-0.43; 0.79] 6.9%
Random effects model -0.04 [-0.31; 0.24] 100.0%
Heterogeneity: I^2 = 85%, τ^2 = 0.1882, p < 0.01
-1.5 -1 -0.5 0 0.5 1 1.5

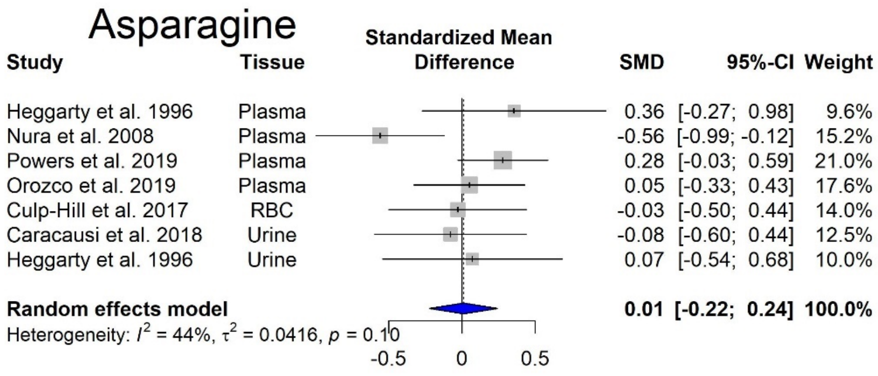

Asparagine
Standardized Mean Difference
Study
Tissue
SMD
95%-CI
Weight
Heggarty et al. 1996 Plasma 0.36 [-0.27; 0.98] 9.6%
Nura et al. 2008 Plasma -0.56 [-0.99; -0.12] 15.2%
Powers et al. 2019 Plasma 0.28 [-0.03; 0.59] 21.0%
Orozco et al. 2019 Plasma 0.05 [-0.33; 0.43] 17.6%
Culp-Hill et al. 2017 RBC -0.03 [-0.50; 0.44] 14.0%
Caracausi et al. 2018 Urine -0.08 [-0.60; 0.44] 12.5%
Heggarty et al. 1996 Urine 0.07 [-0.54; 0.68] 10.0%
Random effects model 0.01 [-0.22; 0.24] 100.0%
Heterogeneity: I^2 = 44%, τ^2 = 0.0416, p = 0.10
-0.5 0 0.5

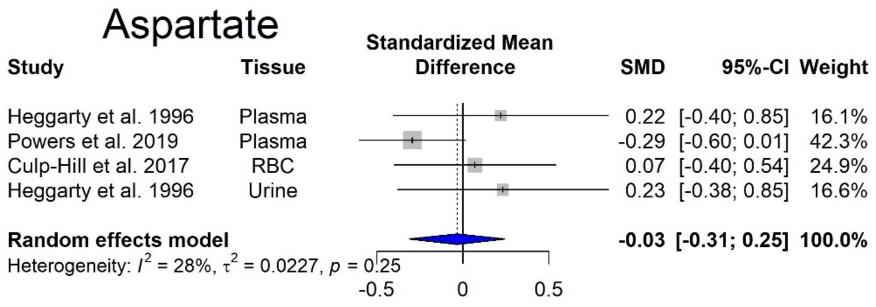

Aspartate
Standardized Mean Difference
Study
Tissue
SMD
95%-CI
Weight
Heggarty et al. 1996 Plasma 0.22 [-0.40; 0.85] 16.1%
Powers et al. 2019 Plasma -0.29 [-0.60; 0.01] 42.3%
Culp-Hill et al. 2017 RBC 0.07 [-0.40; 0.54] 24.9%
Heggarty et al. 1996 Urine 0.23 [-0.38; 0.85] 16.6%
Random effects model -0.03 [-0.31; 0.25] 100.0%
Heterogeneity: I^2 = 28%, τ^2 = 0.0227, p = 0.25
-0.5 0 0.5

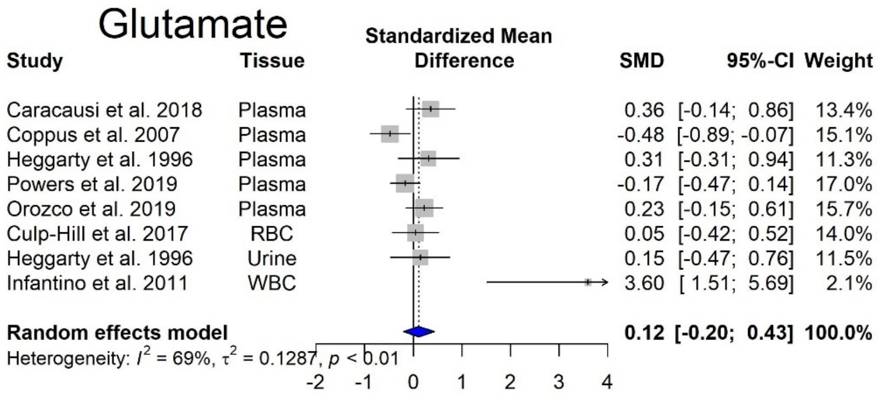

Glutamate
Standardized Mean Difference
Study
Tissue
SMD
95%-CI
Weight
Caracausi et al. 2018 Plasma 0.36 [-0.14; 0.86] 13.4%
Coppus et al. 2007 Plasma -0.48 [-0.89; -0.07] 15.1%
Heggarty et al. 1996 Plasma 0.31 [-0.31; 0.94] 11.3%
Powers et al. 2019 Plasma -0.17 [-0.47; 0.14] 17.0%
Orozco et al. 2019 Plasma 0.23 [-0.15; 0.61] 15.7%
Culp-Hill et al. 2017 RBC 0.05 [-0.42; 0.52] 14.0%
Heggarty et al. 1996 Urine 0.15 [-0.47; 0.76] 11.5%
Infantino et al. 2011 WBC 3.60 [1.51; 5.69] 2.1%
Random effects model 0.12 [-0.20; 0.43] 100.0%
Heterogeneity: I^2 = 69%, τ^2 = 0.1287, p < 0.01
-2 -1 0 1 2 3 4

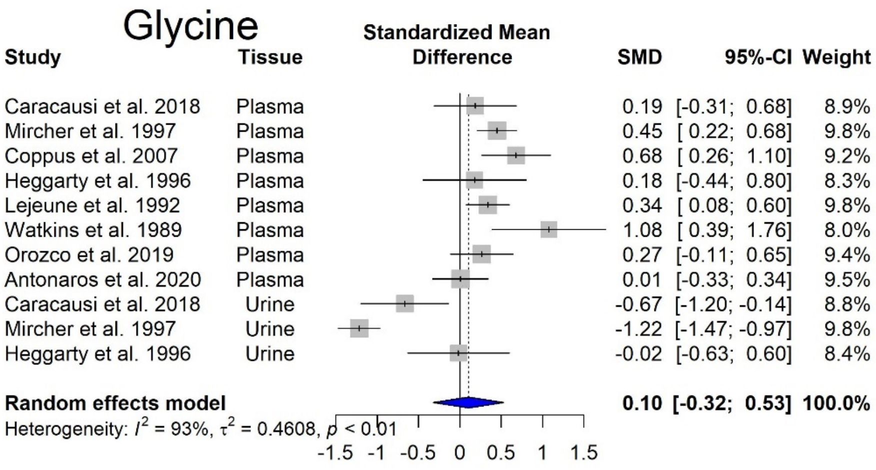
Glycine
Study
Tissue
Standardized Mean Difference
SMD
95%-CI
Weight
Caracausi et al. 2018 Plasma 0.19 [-0.31; 0.68] 8.9%
Mircher et al. 1997 Plasma 0.45 [0.22; 0.68] 9.8%
Coppus et al. 2007 Plasma 0.68 [0.26; 1.10] 9.2%
Heggarty et al. 1996 Plasma 0.18 [-0.44; 0.80] 8.3%
Lejeune et al. 1992 Plasma 0.34 [0.08; 0.60] 9.8%
Watkins et al. 1989 Plasma 1.08 [0.39; 1.76] 8.0%
Orozco et al. 2019 Plasma 0.27 [-0.11; 0.65] 9.4%
Antonaros et al. 2020 Plasma 0.01 [-0.33; 0.34] 9.5%
Caracausi et al. 2018 Urine -0.67 [-1.20; -0.14] 8.8%
Mircher et al. 1997 Urine -1.22 [-1.47; -0.97] 9.8%
Heggarty et al. 1996 Urine -0.02 [-0.63; 0.60] 8.4%
Random effects model 0.10 [-0.32; 0.53] 100.0%
Heterogeneity: I^2 = 93%, τ^2 = 0.4608, $p < 0.01$
-1.5 -1 -0.5 0 0.5 1 1.5

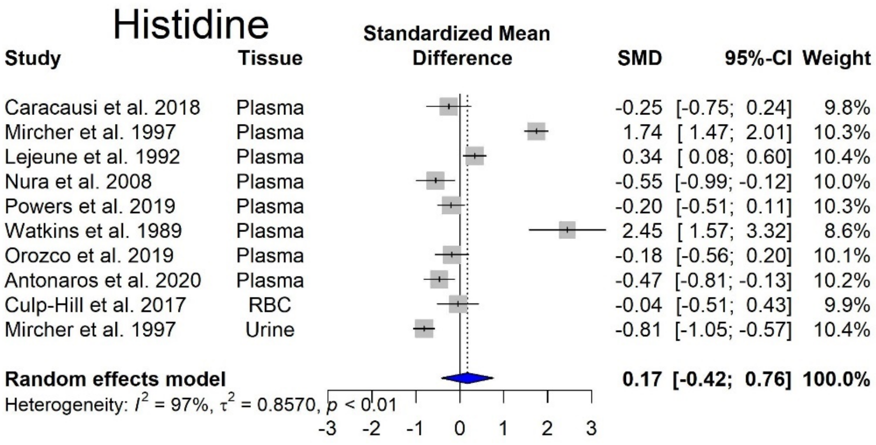
Histidine
Study
Tissue
Standardized Mean Difference
SMD
95%-CI
Weight
Caracausi et al. 2018 Plasma -0.25 [-0.75; 0.24] 9.8%
Mircher et al. 1997 Plasma 1.74 [1.47; 2.01] 10.3%
Lejeune et al. 1992 Plasma 0.34 [0.08; 0.60] 10.4%
Nura et al. 2008 Plasma -0.55 [-0.99; -0.12] 10.0%
Powers et al. 2019 Plasma -0.20 [-0.51; 0.11] 10.3%
Watkins et al. 1989 Plasma 2.45 [1.57; 3.32] 8.6%
Orozco et al. 2019 Plasma -0.18 [-0.56; 0.20] 10.1%
Antonaros et al. 2020 Plasma -0.47 [-0.81; -0.13] 10.2%
Culp-Hill et al. 2017 RBC -0.04 [-0.51; 0.43] 9.9%
Mircher et al. 1997 Urine -0.81 [-1.05; -0.57] 10.4%
Random effects model 0.17 [-0.42; 0.76] 100.0%
Heterogeneity: I^2 = 97%, τ^2 = 0.8570, $p < 0.01$
-3 -2 -1 0 1 2 3

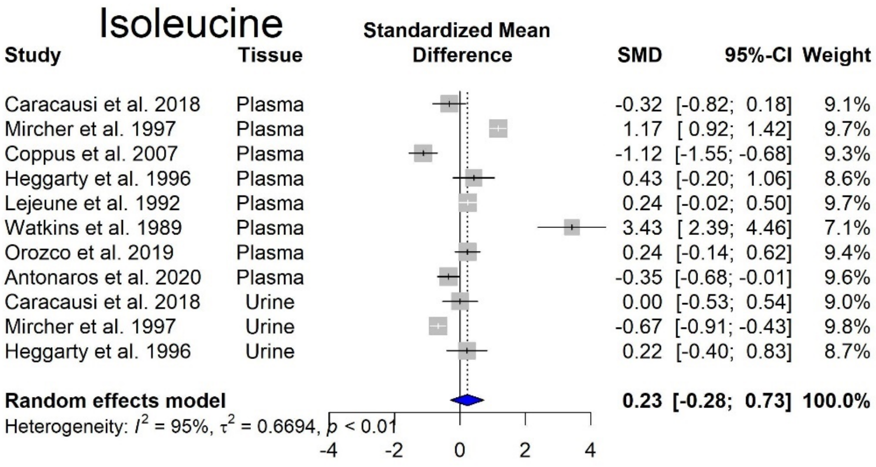
Isoleucine
Study
Tissue
Standardized Mean Difference
SMD
95%-CI
Weight
Caracausi et al. 2018 Plasma -0.32 [-0.82; 0.18] 9.1%
Mircher et al. 1997 Plasma 1.17 [0.92; 1.42] 9.7%
Coppus et al. 2007 Plasma -1.12 [-1.55; -0.68] 9.3%
Heggarty et al. 1996 Plasma 0.43 [-0.20; 1.06] 8.6%
Lejeune et al. 1992 Plasma 0.24 [-0.02; 0.50] 9.7%
Watkins et al. 1989 Plasma 3.43 [2.39; 4.46] 7.1%
Orozco et al. 2019 Plasma 0.24 [-0.14; 0.62] 9.4%
Antonaros et al. 2020 Plasma -0.35 [-0.68; -0.01] 9.6%
Caracausi et al. 2018 Urine 0.00 [-0.53; 0.54] 9.0%
Mircher et al. 1997 Urine -0.67 [-0.91; -0.43] 9.8%
Heggarty et al. 1996 Urine 0.22 [-0.40; 0.83] 8.7%
Random effects model 0.23 [-0.28; 0.73] 100.0%
Heterogeneity: I^2 = 95%, τ^2 = 0.6694, $p < 0.01$
-4 -2 0 2 4

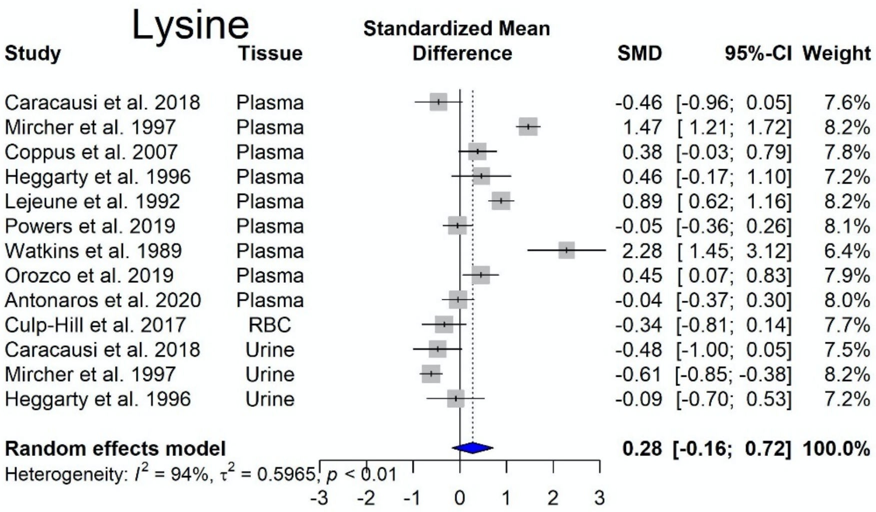
Lysine
Study Tissue Standardized Mean Difference SMD 95%-CI Weight
Caracausi et al. 2018 Plasma -0.46 [-0.96; 0.05] 7.6%
Mircher et al. 1997 Plasma 1.47 [1.21; 1.72] 8.2%
Coppus et al. 2007 Plasma 0.38 [-0.03; 0.79] 7.8%
Heggarty et al. 1996 Plasma 0.46 [-0.17; 1.10] 7.2%
Lejeune et al. 1992 Plasma 0.89 [0.62; 1.16] 8.2%
Powers et al. 2019 Plasma -0.05 [-0.36; 0.26] 8.1%
Watkins et al. 1989 Plasma 2.28 [1.45; 3.12] 6.4%
Orozco et al. 2019 Plasma 0.45 [0.07; 0.83] 7.9%
Antonaros et al. 2020 Plasma -0.04 [-0.37; 0.30] 8.0%
Culp-Hill et al. 2017 RBC -0.34 [-0.81; 0.14] 7.7%
Caracausi et al. 2018 Urine -0.48 [-1.00; 0.05] 7.5%
Mircher et al. 1997 Urine -0.61 [-0.85; -0.38] 8.2%
Heggarty et al. 1996 Urine -0.09 [-0.70; 0.53] 7.2%
Random effects model 0.28 [-0.16; 0.72] 100.0%
Heterogeneity: I^2 = 94%, τ^2 = 0.5965, p < 0.01
-3 -2 -1 0 1 2 3

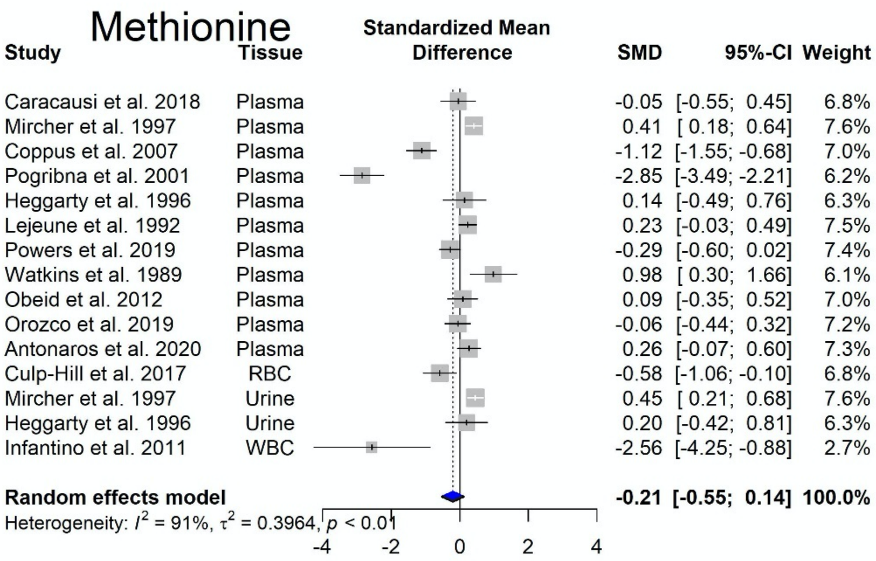
Methionine
Study Tissue Standardized Mean Difference SMD 95%-CI Weight
Caracausi et al. 2018 Plasma -0.05 [-0.55; 0.45] 6.8%
Mircher et al. 1997 Plasma 0.41 [0.18; 0.64] 7.6%
Coppus et al. 2007 Plasma -1.12 [-1.55; -0.68] 7.0%
Pogribna et al. 2001 Plasma -2.85 [-3.49; -2.21] 6.2%
Heggarty et al. 1996 Plasma 0.14 [-0.49; 0.76] 6.3%
Lejeune et al. 1992 Plasma 0.23 [-0.03; 0.49] 7.5%
Powers et al. 2019 Plasma -0.29 [-0.60; 0.02] 7.4%
Watkins et al. 1989 Plasma 0.98 [0.30; 1.66] 6.1%
Obeid et al. 2012 Plasma 0.09 [-0.35; 0.52] 7.0%
Orozco et al. 2019 Plasma -0.06 [-0.44; 0.32] 7.2%
Antonaros et al. 2020 Plasma 0.26 [-0.07; 0.60] 7.3%
Culp-Hill et al. 2017 RBC -0.58 [-1.06; -0.10] 6.8%
Mircher et al. 1997 Urine 0.45 [0.21; 0.68] 7.6%
Heggarty et al. 1996 Urine 0.20 [-0.42; 0.81] 6.3%
Infantino et al. 2011 WBC -2.56 [-4.25; -0.88] 2.7%
Random effects model -0.21 [-0.55; 0.14] 100.0%
Heterogeneity: I^2 = 91%, τ^2 = 0.3964, p < 0.01
-4 -2 0 2 4

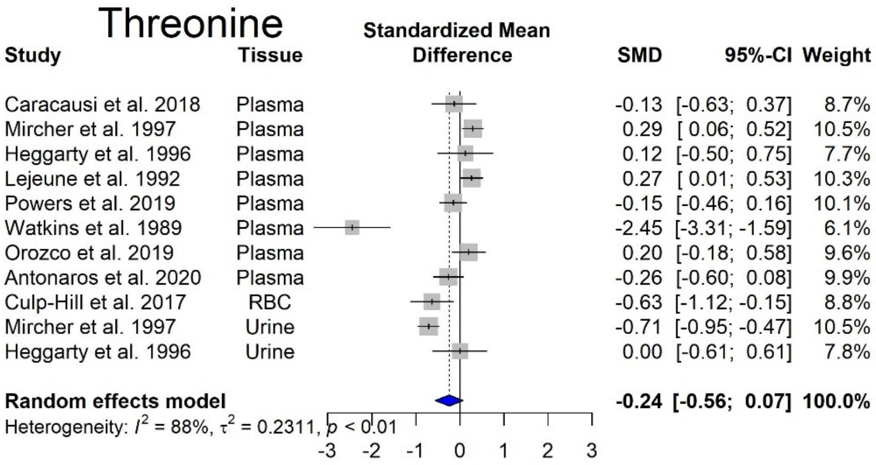
Threonine
Study Tissue Standardized Mean Difference SMD 95%-CI Weight
Caracausi et al. 2018 Plasma -0.13 [-0.63; 0.37] 8.7%
Mircher et al. 1997 Plasma 0.29 [0.06; 0.52] 10.5%
Heggarty et al. 1996 Plasma 0.12 [-0.50; 0.75] 7.7%
Lejeune et al. 1992 Plasma 0.27 [0.01; 0.53] 10.3%
Powers et al. 2019 Plasma -0.15 [-0.46; 0.16] 10.1%
Watkins et al. 1989 Plasma -2.45 [-3.31; -1.59] 6.1%
Orozco et al. 2019 Plasma 0.20 [-0.18; 0.58] 9.6%
Antonaros et al. 2020 Plasma -0.26 [-0.60; 0.08] 9.9%
Culp-Hill et al. 2017 RBC -0.63 [-1.12; -0.15] 8.8%
Mircher et al. 1997 Urine -0.71 [-0.95; -0.47] 10.5%
Heggarty et al. 1996 Urine 0.00 [-0.61; 0.61] 7.8%
Random effects model -0.24 [-0.56; 0.07] 100.0%
Heterogeneity: I^2 = 88%, τ^2 = 0.2311, p < 0.01
-3 -2 -1 0 1 2 3

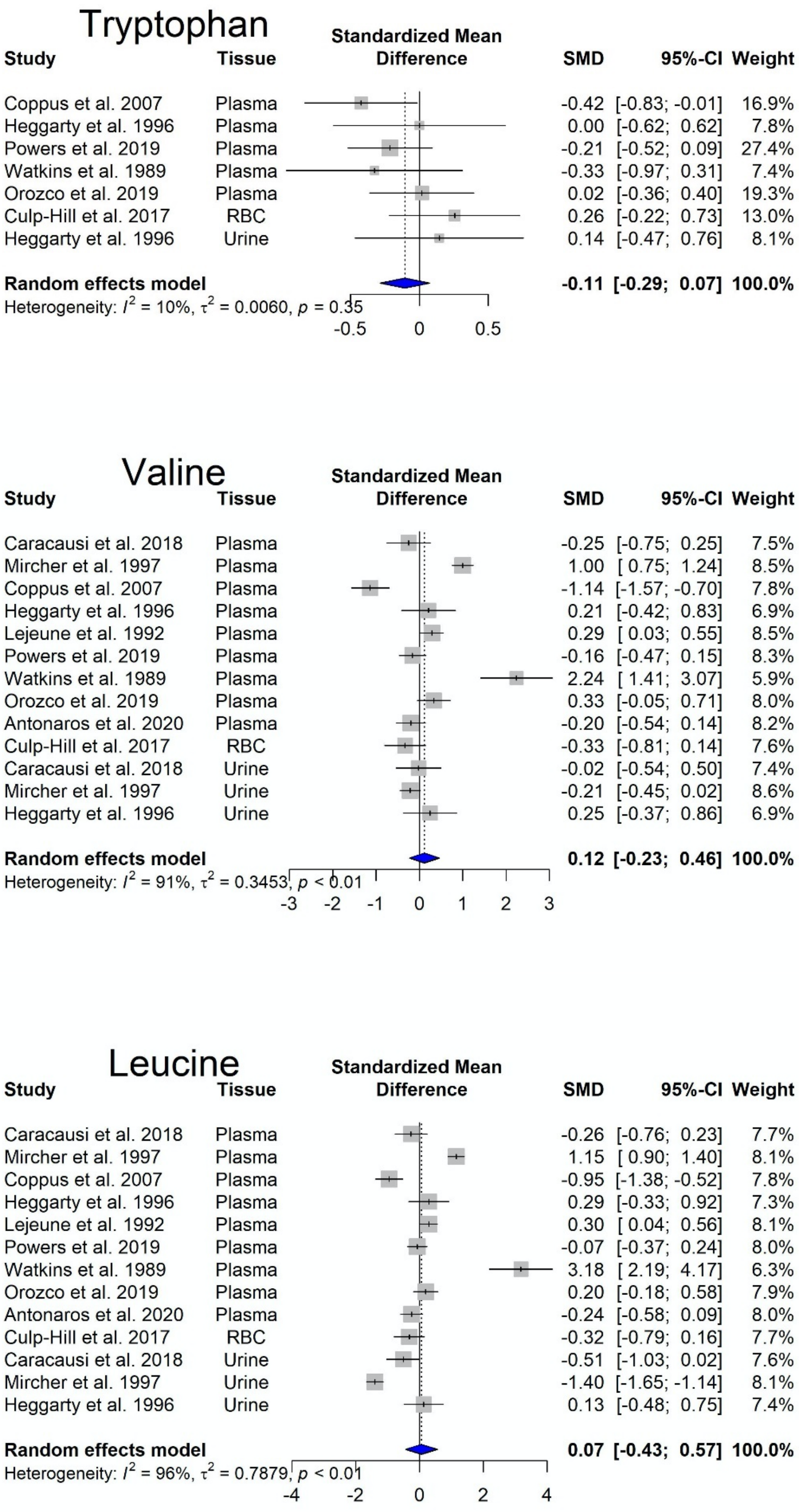

Supplementary Fig. 9. Meta-analysis of amino acids not included in the Main Text.

References

Antonaros, F., Ghini, V., Pulina, F., Ramacieri, G., Cicchini, E., Mannini, E., Martelli, A., Feliciello, A., Lanfranchi, S., Onnivello, S., Vianello, R., Locatelli, C., Cocchi, G., Pelleri, M. C., Vitale, L., Strippoli, P., Luchinat, C., Turano, P., Piovesan, A., and Caracausi, M. (2020) Plasma metabolome and cognitive skills in Down syndrome. *Scientific Reports* **10**, 10491.

Atkinson, D.E., Walton, G.M. (1967) Adenosine triphosphate conversion in metabolic regulation. Rat liver cleavage enzyme. *The Journal of Biological Chemistry* **242,** 3239-3241.

Caracausi, M., Ghini, V., Locatelli, C., Mericio, M., Piovesan, A., Antonaros, F., Pelleri, M. C., Vitale, L., Vacca, R. A., and Bedetti, F. (2018) Plasma and urinary metabolomic profiles of Down syndrome correlate with alteration of mitochondrial metabolism. *Scientific reports* **8**, 1-16.

Culp-Hill, R., Zheng, C., Reisz, J. A., Smith, K., Rachubinski, A., Nemkov, T., Butcher, E., Granrath, R., Hansen, K. C., and Espinosa, J. M. (2017) Red blood cell metabolism in Down syndrome: Hints on metabolic derangements in aging. *Blood advances* **1**, 2776-2780.

Dieterle, F., Ross, A., Schlotterbeck, G., and Senn, H. (2006) Probabilistic quotient normalization as robust method to account for dilution of complex biological mixtures. Application in 1H NMR metabonomics. *Anal Chem* **78**, 4281-4290.

Huber, W., von Heydebreck, A., Sültmann, H., Poustka, A., and Vingron, M. (2002) Variance stabilization applied to microarray data calibration and to the quantification of differential expression. *Bioinformatics* **18 Suppl 1**, S96-104.

Powers, R. K., Culp-Hill, R., Ludwig, M. P., Smith, K. P., Waugh, K. A., Minter, R., Tuttle, K. D., Lewis, H. C., Rachubinski, A. L., and Granrath, R. E. (2019) Trisomy 21 activates the kynurenine pathway via increased dosage of interferon receptors. *Nature communications* **10**, 1-11.

Ritchie, M. E., Phipson, B., Wu, D., Hu, Y., Law, C. W., Shi, W., and Smyth, G. K. (2015) Limma powers differential expression analyses for RNA-sequencing and microarray studies. *Nucleic Acids Res* **43**, e47.
